# Giant Thermosalient Effect in a Molecular Single Crystal: Dynamic Transformations and Mechanistic Insights

**DOI:** 10.1021/jacs.4c09222

**Published:** 2024-09-24

**Authors:** Mohammad Afsar Uddin, Raúl Martín, Sergio Gámez-Valenzuela, Marcelo Echeverri, M. Carmen Ruiz Delgado, Enrique Gutiérrez Puebla, Angeles Monge, Berta Gómez-Lor

**Affiliations:** † Instituto de Ciencia de Materiales de Madrid, CSIC, Cantoblanco, 28049 Madrid, Spain; ‡ Faculty of Chemical and Technologies Sciences, University of Castilla-La Mancha, 13071 Ciudad Real, Spain; § Department of Physical Chemistry, University of Málaga, Campus de Teatinos s/n, 29071 Málaga, Spain

## Abstract

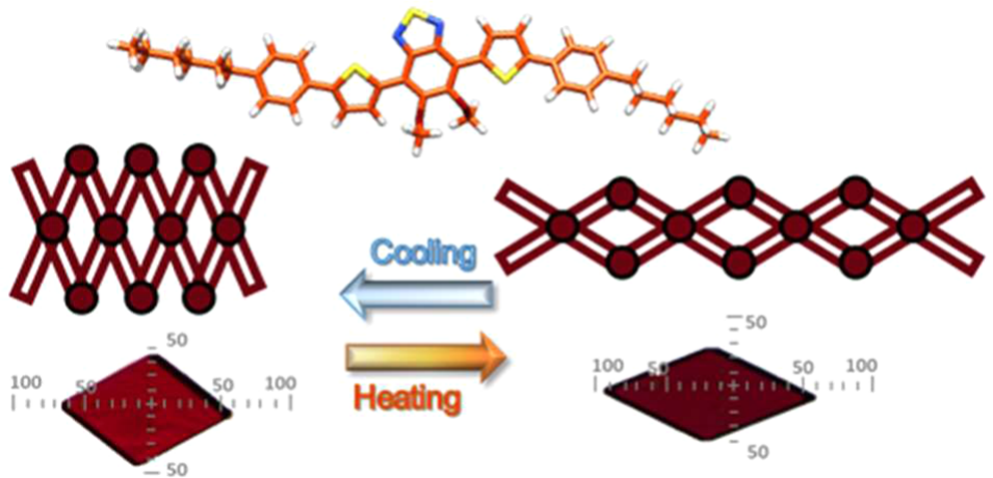

The exploration of mechanical motion in molecular crystals under external stimuli is of great interest because of its potential applications in diverse fields, such as electronics, actuation, or sensing. Understanding the underlying processes, including phase transitions and structural changes, is crucial for exploiting the dynamic nature of these crystals. Here, we present a novel organic compound, **PT-BTD**, consisting of five interconnected aromatic units and two peripheral alkyl chains, which forms crystals that undergo a drastic anisotropic expansion (33% in the length of one of its dimensions) upon thermal stimulation, resulting in a pronounced deformation of their crystal shape. Remarkably, the transformation occurs while maintaining the single-crystal nature, which has allowed us to follow the crystal-to-crystal transformation by single-crystal analysis of the initial and expanded polymorphs, providing valuable insights into the underlying mechanisms of this unique thermosalient behavior. At the molecular level, this transformation is associated with subtle, coordinated conformational changes, including slight rotations of the five interconnected aromatic units in its structure and increased dynamism in one of its peripheral alkyl chains as the temperature rises, leading to the displacement of the molecules. In situ polarized optical microscopy reveals that this transformation occurs as a rapidly advancing front, indicative of a martensitic phase transition. The results of this study highlight the crucial role of a soft and flexible structural configuration combined with a highly compact but loosely bound supramolecular structure in the design of thermoelastic materials.

## Introduction

Stimuli-responsive organic materials, whose properties can be dynamically modulated in a controlled manner by external triggers to perform specific tasks, have attracted enormous interest.^1−5^ In this field, stimuli-responsive polymorphic single-crystalline materials are of particular interest because they allow the investigation of the detailed mechanisms underlying their smart behavior, providing guidelines for the future fabrication and development of new smart organic crystals and their applications.^5−10^ The task of imparting stimuli-responsive properties to a single crystal can be approached by inducing conformational changes in the constituting molecular units.^11,12^ Alternatively, taking advantage of the collective nature of crystal properties and therefore their high sensitivity to how molecules interact with each other, efforts can be focused on inducing variations in crystal packing by breaking and restoring weak intermolecular interactions upon external stimulation.^6,13−16^

Although generally organic single-crystal materials are brittle, several organic crystals that withstand mechanical stress and deformation without breaking have recently been reported.^8,17−20^ Such exceptional mechanical flexibility arouses much interest in device applications where durability and mechanical robustness are critical factors.^9,21−23^

In fact, there is a continuously growing number of crystals that show the mechanical motion of molecular crystals upon exposure to external stimuli. Some molecular crystals, when subjected to thermal stimuli, cross a critical phase transition state, in which the cooperative movement of molecules may lead to a sudden change in shape and/or volume, causing the crystal to “jump” or exhibit visible mechanical motion.^24,25^ This phenomenon, commonly referred to as the thermosalient effect,^26−28^ is characterized by the crystal undergoing an abrupt and visible change in its shape or position upon exposure to heating or cooling stimuli. The extent of movement and specific behavior vary depending on the molecular structure, symmetry, and interactions within the crystal lattice.

While this effect has long been observed in inorganic crystals, large thermal expansion effects in organic single crystals are significantly less common.^29^ The thermosalient behavior in organic single crystals has been successfully utilized in precisely controlled switchable electronics,^21,30^ ferroelectrics,^31^ actuators,^32−35^ sensing,^36^ and electromechanical devices,^37^ with potential applications in soft robotics, nanomachines, or smart adaptive systems. However, to advance to real-life applications, it is crucial to understand the underlying mechanisms of this unique behavior. Although the discovery of organic thermosalient crystals has largely been serendipitous at this stage, researchers are making significant efforts to elucidate the structural features responsible for thermosalient behavior.^29^

**Figure 1:**
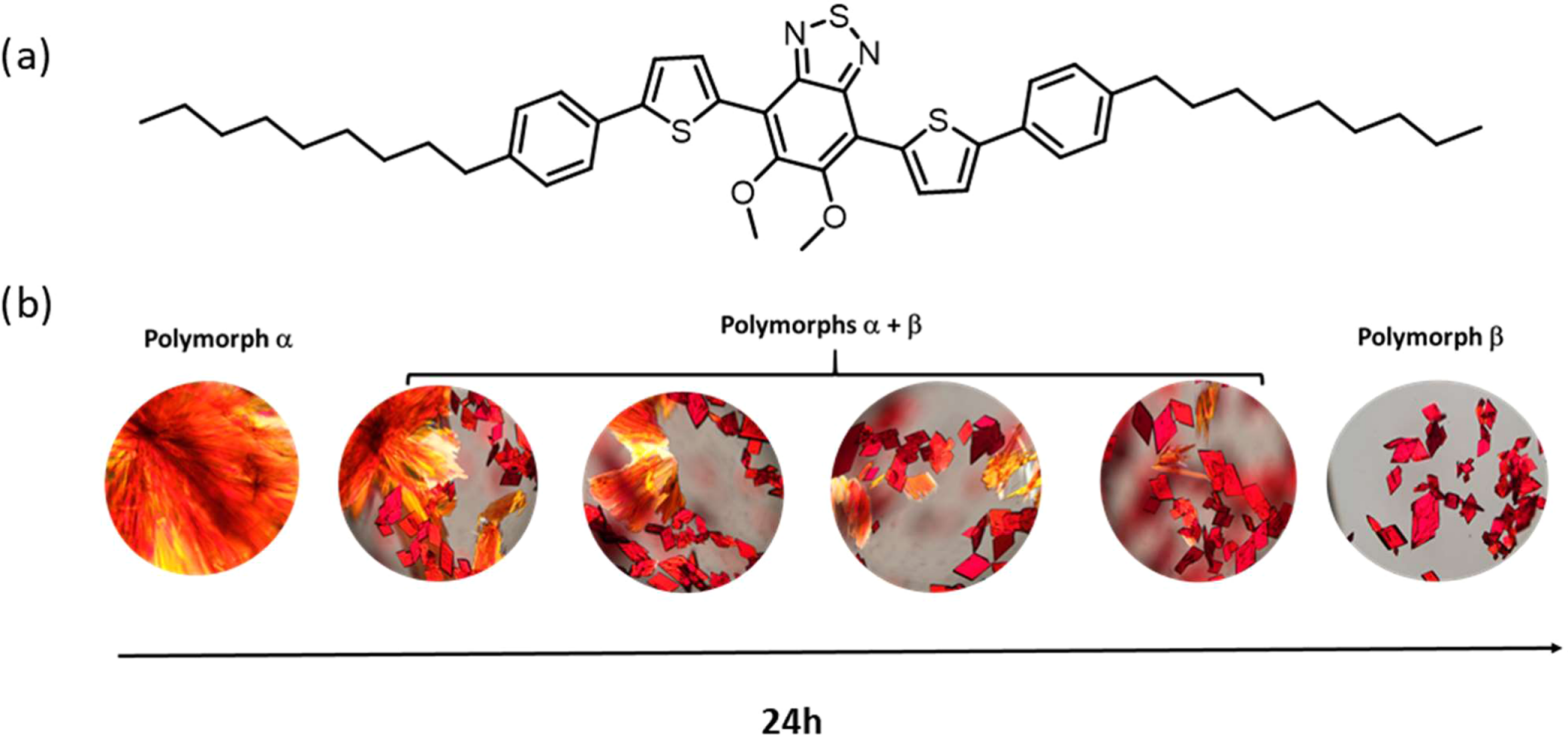
(a) Chemical structure of **PT-BTD**. (b) Evolution of the crystallization of **PT-BTD** visualized under a polarizing optical microscope by diffusion of MeOH vapors into a THF solution of the compound, showing the transformation of polymorph **α** into **β**.

In this article, we present a new rod-shaped molecule 4,7-bis[5-(4-nonylphenyl)-2-thienyl]-5,6-dimethoxy-2,1,3-benzothiadiazole **PT-BTD** (Figure 1a) which, upon thermal stimulation, exhibits high anisotropic lattice expansions and reversible crystal shape deformation, which translates in a thermosalient behavior.

The molecular structure of **PT-BTD** consists of five interconnected aromatic units (a 2,3-methoxybenzothiadiazole symmetrically substituted at positions 4 and 7 with two thiophene rings linked to two terminal phenyl rings), flanked by two flexible nonyl chains to induce its arrangement in layered structures and to favor the emergence of low interacting slip planes that can facilitate gliding processes. Note that benzothiadiazole forms relatively directional weak interactions^38^ that can be easily disrupted in favor of other intermolecular interactions^38,39^ making it very interesting in the search for stimuli-responsive materials.^6,40−42^ The flexibility of the conjugated five-ring structure and the methoxy functionalities increase the degrees of rotational freedom and give the molecule the capacity to adopt close, energetically stable conformers. This structural design confers a rich polymorphism to this molecule.

A comprehensive analysis involving single-crystal X-ray diffraction (SCXRD), Raman spectroscopy, and theoretical calculations has contributed to rationalize the rich polymorphism shown by this material, highlighting the crucial role of a soft and flexible structural configuration, combined with a highly compact but loosely bound supramolecular structure, in the development of thermoelastic materials.

## Results and Discussion

### Synthesis and Crystal Growth of PT-BTD

**PT-BTD** was synthesized through the Suzuki cross-coupling reaction of 4,7-bis(5-bromo-2-thienyl)-5,6-dimethoxy-2,1,3-benzothiadiazole^43,44^ with 2 equiv of 4-nonylphenylboronic acid. After purification by column chromatography using hexane/CH_2_Cl_2_ (3:1) as the eluent, **PT-BTD** was obtained as a red, highly fluorescent solid in 65% yield. The optical properties of **PT-BTD** were initially investigated in solution by UV–vis absorption and fluorescence spectroscopy (Figure S1). **PT-BTD** displays distinctive absorption bands at 325 and 482 nm in a 10^–5^ M CH_2_Cl_2_ solution attributed to the benzothiadiazole unit (BTD). A charge-transfer transition between the moderate donor thienyl**-**alkylphenyl group and the strong electron acceptor BTD moiety is responsible for the lowest energy absorption band, as demonstrated by time-dependent-density-functional theory (TD-DFT) calculations (see Supporting Information Figure S23). The fluorescence spectrum of **PT-BTD** in CH_2_Cl_2_ solution is characterized by an emission band with a peak at 629 nm under excitation at 475 nm, with quantum yields as high as 75%, revealing the attractive fluorescence properties of this compound.

Attempts to crystallize **PT-BTD** by slow evaporation of a CH_2_Cl_2_ solution resulted in a polycrystalline red solid, in which visualization under a UV lamp (320 nm) revealed the presence of two polymorphs emitting different colors (orange and red). We were able to selectively grow high-quality crystals of both polymorphs suitable for crystal structure determination by the vapor diffusion method, in which nonsolvent MeOH vapors were diffused into a THF solution of the compound.

Under these conditions, initially, orange thin crystals emitting at 646 nm (polymorph **α**) were obtained. However, curiously, as the THF solution is gradually enriched in MeOH, red-rhombus-shaped crystals were formed (polymorph **β**) that emitted at 667 nm. Interestingly, similar photoluminescence quantum yields (Φ_F_ = 22.3% for **α** and Φ_F_ = 17.2% for **β**) and lifetimes (τ_F_ ≈ 7–9 ns) are observed for both polymorphs (Figures S3 and S4). Figure 1 shows the evolution of the crystallization process of **PT-BTD** with time. This transformation can be understood as a solution-crystallization process. The purity of the initial and the transformed polymorphs could be confirmed by powder X-ray diffraction of the corresponding bulk material, in both cases characterized by an intense low angle reflection at 2θ = 2.82° for **α** and 2θ = 4.18° for **β** (see Figure S5).

### Thermal Properties

Thermal analysis of the two polymorphs was studied through thermogravimetric analysis (TGA) and differential scanning calorimetry (DSC). TGA shows that **PT-BTD** is stable above 300 °C (Figure S6). DSC shows a rich polymorphism and evidence that after melting and subsequent cooling, polymorph **β** transforms again into polymorph **α**.

**Figure 2:**
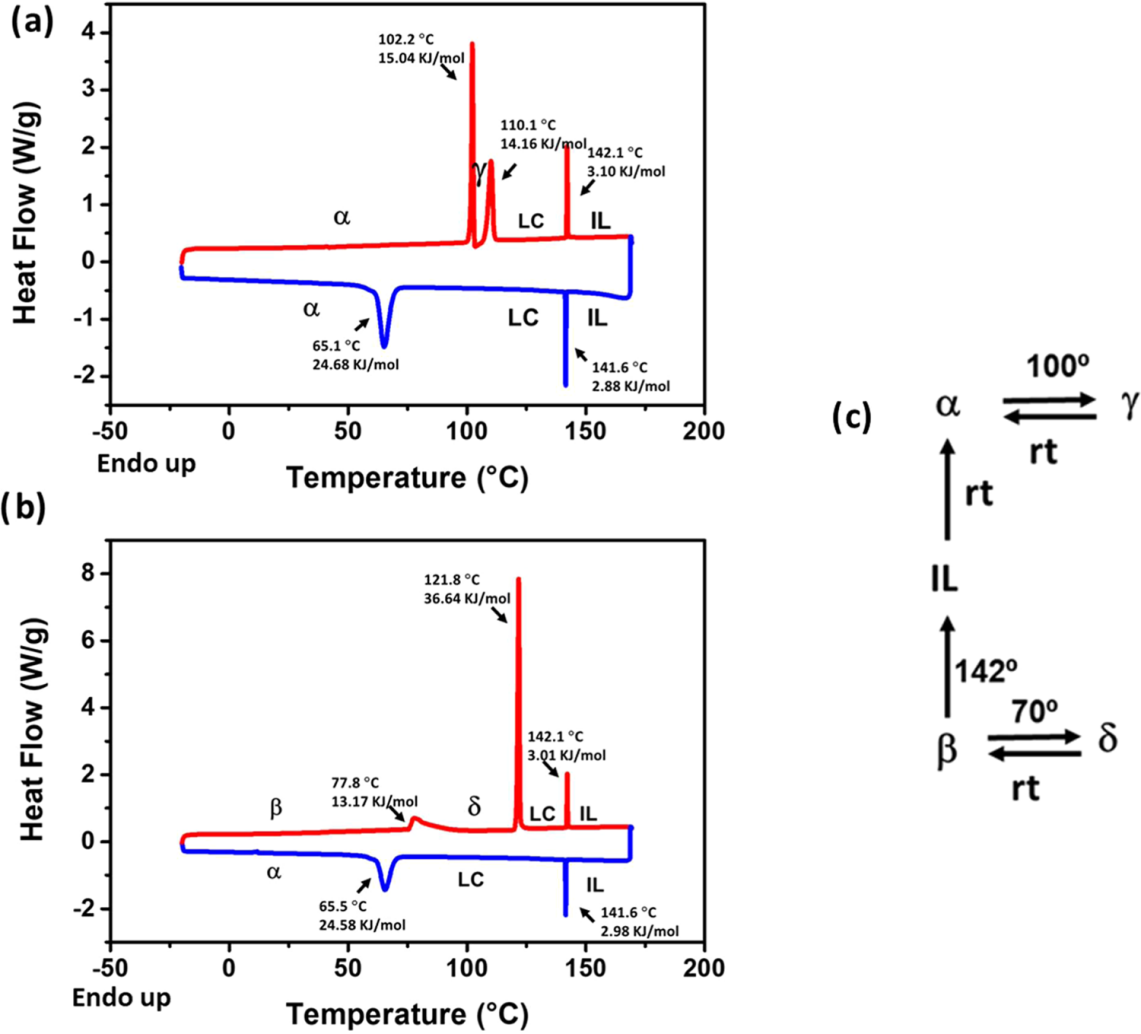
DSC first heating (red) and cooling (blue) cycles of polymorphs (a) **α** and (b) **β**. DSC cycles were recorded at 10 °C/min. Endo up. (c) Schematic representation of the thermal polymorphic transformations.

When heated, polymorph **α** experiments a crystal-to-crystal transformation at 101 °C to yield polymorph **γ**, followed by a crystal-to-mesophase transformation at 121 °C, prior to melting at 142 °C (Figure 2a). On the cooling cycle, the material transitions into a smectic C mesophase (see Figure S11), covering a temperature range from 141 to 65 °C, before crystallizing back into the initial polymorph **α**. This behavior is reproduced in subsequent cycles.

In contrast, polymorph **β** exhibits distinct thermal behavior during the first heating cycle. It undergoes a crystal-to-crystal transformation at 78 °C (polymorph **β** to polymorph **δ**), followed by a crystal-to-liquid crystal (**LC**) mesophase transformation at 120 °C, before melting (**IL**) at 141 °C. Upon cooling back to room temperature, the material reverts to a mesophase, spanning the temperature range from 141 to 69 °C, before crystallizing (Figure 2b). However, this thermal behavior is not reproduced in the following cycles. Conversely, the subsequent heating–cooling cycles replicate the behavior observed during the thermal analysis of polymorph **α** (Figure S7), indicating that after the first cooling, polymorph **β** transforms again into polymorph **α**, as could be confirmed by powder X-ray diffraction (Figure S19a). Interestingly, if during the first heating cycle, polymorph **β** does not reach the crystal-to-mesophase temperature (120 °C), the transformation from polymorph **β** to polymorph **δ** remains reversible over multiple cycles (Figure S10).

These complex polymorphic transformations were initially traced by Raman spectroscopy (see the Supporting Information). Note that Raman spectroscopy has previously demonstrated its utility in providing accurate intermolecular and intramolecular structural information during the polymorphic transitions of BTD derivatives and other π-conjugated systems.^6,45,40,46,47^

**Figure 3:**
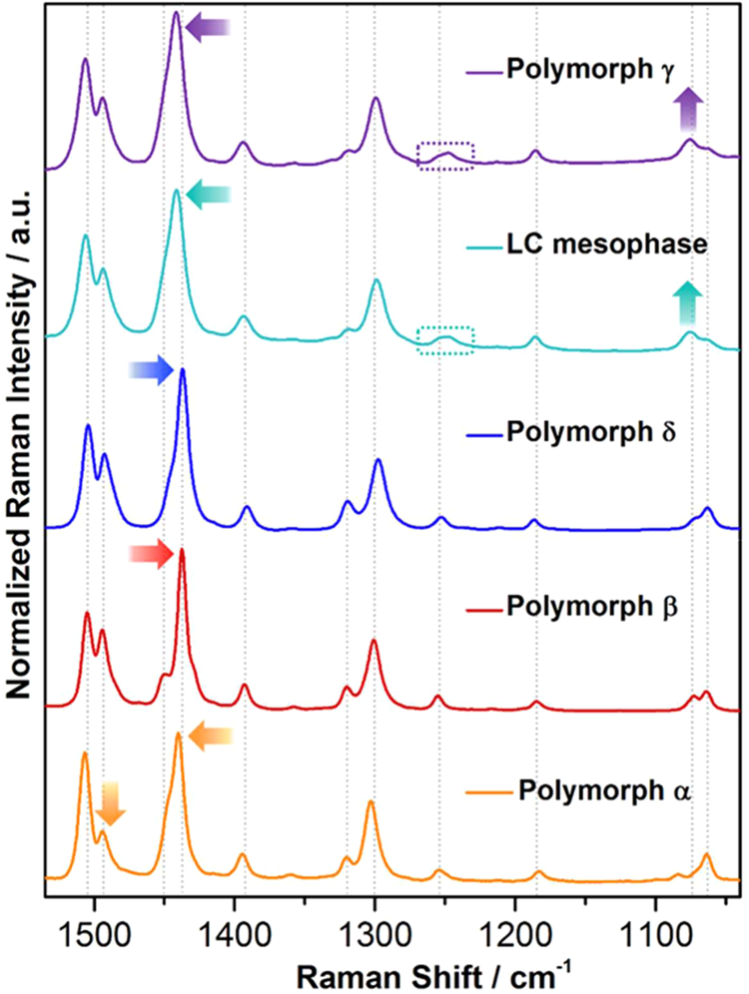
Comparison between the Raman spectra of all of the studied phases collected at 785 nm excitation.

Figure 3 shows the Raman spectra for the different phases, highlighting the most significant changes. The most prominently affected bands are (i) the main Raman band at 1450 cm^–1^, assigned to collective C–C/C=C stretching vibrations mainly involving the thiophene rings and the BTD unit; (ii) the band at 1494 cm^–1^, associated with C–C and C–N stretching vibrations within the BTD unit; (iii) the band at 1250 cm^–1^, linked to C–C stretching bonds between rings; and (iv) the bands at 1064 and 1073 cm^–1^, assigned to the trans (ν(C–C)_T_) and gauche (ν(C–C)_G_) isomers of the alkyl chains, respectively. The vibrational eigenvectors associated with the most outstanding C=C/C–C Raman features can be found in Figure S32.

The changes observed across the different phases are indicative of distinct structural and conformational characteristics. In polymorphs **β** and **δ**, the main Raman band at 1450 cm^–1^ shifts to lower frequencies, pointing to increased distortion of the thiophene rings relative to the BTD unit. On the other hand, polymorph **α** shows a reduced intensity of the 1494 cm^–1^ band, suggesting alterations in the supramolecular arrangement of the BTD moiety. Finally, both the **LC** mesophase and polymorph **γ** exhibit a broadening of the 1250 cm^–1^ band and a change in the intensity ratio between the 1064 and 1073 cm^–1^ peaks, reflecting a high degree of conformational disorder. A follow-up of the complex polymorphic transformation of **PT-BTD** upon heating and a more detailed description of the Raman spectral changes can be found in the Supporting information.

### Single-Crystal Structure Determination

**Figure 4:**
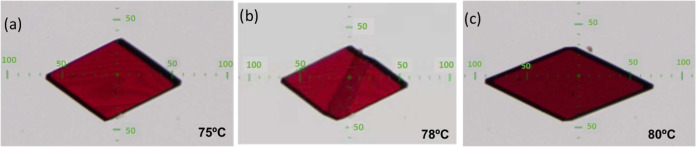
Microphotographs of a single crystal of **PT-BTD** (polymorph **β**) before (a), during (b), and after (c) thermal expansion showing its transformation to polymorph **δ** captured using an optical microscope equipped with a Linkam hot stage. The scale units are microns.

In an attempt to get a deeper insight into the thermal transformations of polymorphs **α** into **γ** and **β** into **δ**, single crystals of **α** and **β**, suitable for X-ray structure analysis, were selected and heated at the temperature of the phase transitions under an optical microscope equipped with a heating stage. Interestingly, while the thermal transformation of **α** into **γ** polymorph produces cracks in the crystal, which eventually leads to its breakage, the transition of the rhombohedral red polymorph **β** into **δ** taking place at 78 °C maintains the single-crystal integrity, despite occurring with a clear shape deformation caused by an anisotropic thermal expansion (Figure 4). In fact, during this single-crystal-to-single-crystal phase transition, a drastic expansion (33% in length) of one of the diagonals of the rhombus, together with a slight contraction with its orthogonal axis, takes place. To our knowledge, this significant shape modification represents the largest mechanical response observed in thermally dynamic single crystals of one-component molecular materials (Table S1). As can be observed in Figure 4b and Video S1, the transformation begins at a midpoint and propagates two-dimensionally, showing a clear phase front sweep characteristic of a martensitic transition.^29,48^ It should be noted that polymorph **β** is highly stable in harsh conditions (Figure S12) and that as long as the temperature is maintained between 78 and 100 °C, the shape of the polymorph **δ** remains unchanged for several hours (Figure S13). When cooling back, the crystal returns to its original shape, displaying thermosalient characteristics by appearing to “jump”. This behavior suggests that the thickness of the crystal contracts upon heating (undergoes negative thermal expansion) and then rapidly expands upon returning to its initial state, leading to the sudden jump and, on occasion, crystal breakage, probably attributed to the shock of the fall (Video S2 and Figure S20).

Remarkably, the fact that the transformation between polymorphs **β** and **δ** takes place while retaining the single-crystal nature has allowed us to determine the structure of the two polymorphs on the same crystal.

Single-crystal structure determination shows that polymorph **α** crystallizes in the *P*2_1_/*c* space group with only one independent molecule in the unit cell (Figure S14a). In this structure, some fractional disordered MeOH molecules were located among the long alkyl chains. In contrast, polymorph **β** crystallizes in the triclinic P-1 space group and contains two independent molecules per unit cell (Figure S14b). Upon heating **β**, the two independent molecules converge to one unique molecule in the asymmetric unit in polymorph **δ**, which crystallizes in the monoclinic *P*2_1_/*c* space group (Figure S14c).

**Figure 5:**
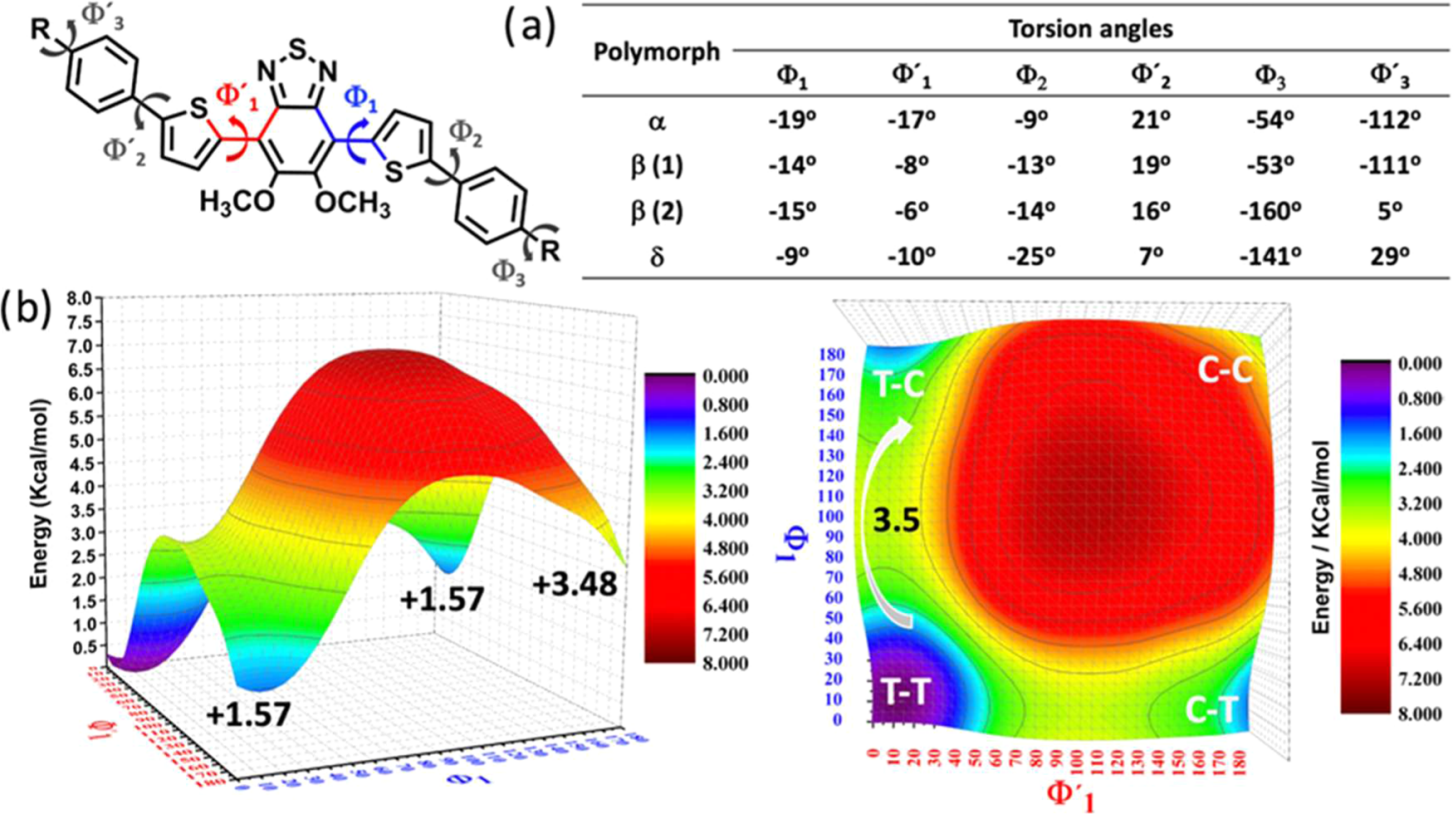
(a) Different torsion angles shown by the independent molecules of **PT-BTD** in polymorphs **α**, **β**, and **δ**, showing variation in the representative angles. (b) Dihedral potential energy hypersurface *E* = *E*(ϕ_1_,ϕ_2_) for an isolated **PT-BTD** molecule calculated at the ωB97XD/6-31G** level of theory.

In all three polymorphs, the two ortho-methoxy groups in the BTD moiety are positioned one above and the other below the plane of the BTD ring, and the thiophene rings assume a *cis*–*trans* configuration with respect to the BTD (Figure 5a). This is in line with the sweep of the potential energy surface of these two ring rotations (Figure 5b), which show that the *cis*–*trans* and *trans*–*trans* configurations have similar energies with an activation barrier of 3.5 kcal/mol. Low potential energy barriers are also found for the rotation between the phenyl and thienyl rings (Figure S22), further supporting the large flexibility of the **PT-BTD** system.

In fact, the crystal structure analysis reveals that the main differences among the independent molecules of the three polymorphs rely on the torsion angles between the five aromatic moieties, varying within the range of 3–25°. Polymorph **α** has the most distorted conjugated backbone, resulting in lower intramolecular electronic communication between the electron-deficient central BTD moiety and the electron-rich thienyl rings, which likely contributes to the blueshift of its absorption and emission color. Large differences in the disposition of the alkyl chains are also observed.

**Figure 6:**
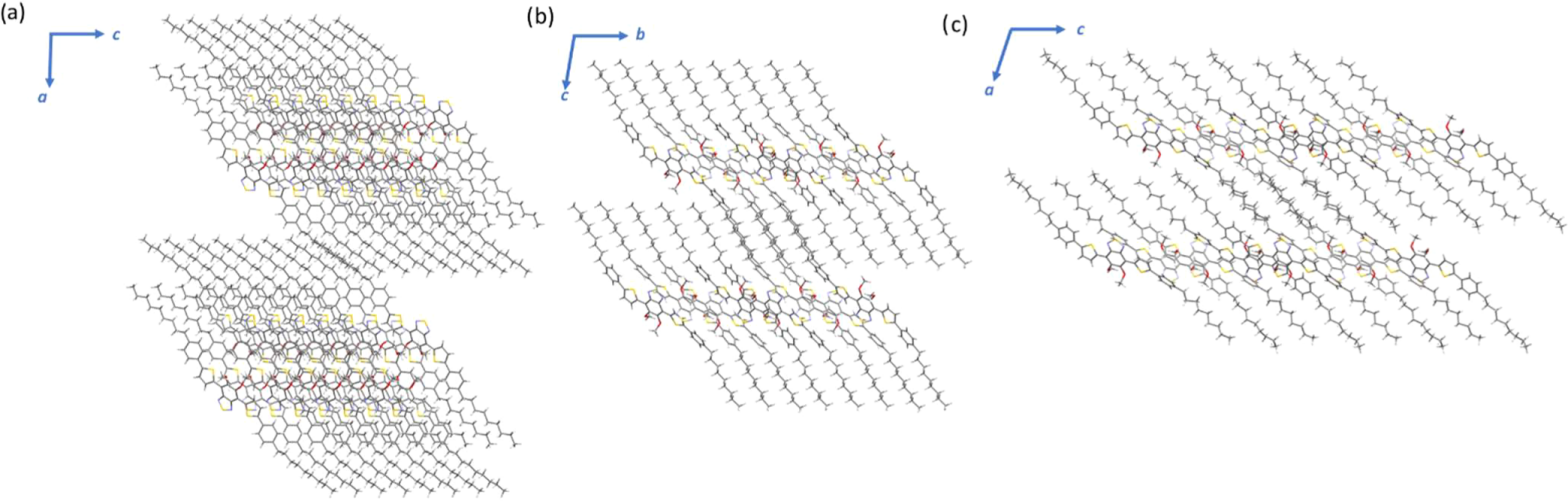
Lamellar arrangement of polymorphs **α**, **β**, and **δ** of **PT-BTD** viewed along *b*-, *a-*, and b-axes, respectively.

As observed in Figure 6, all three polymorphs adopt a lamellar arrangement in which the alkyl chains are strongly interdigitated. In polymorphs **β** and **δ**, with interlayer distances of 21.90 and 17.98 Å, respectively, the repeating unit within the layer consists of individual structural units.

In contrast, polymorph **α** features layers formed by pairs of molecules, resulting in a significantly larger interlayer distance (31.73 Å).

### Elucidation of the Thermosalient Behavior of PT-BTD

To elucidate the mechanism behind the remarkable shape transformation and thermosalient behavior of **PT-BTD**, we analyzed the changes in conformation and molecular packing underlying the thermomechanical martensitic transition. Single-crystal X-ray diffraction (SCXRD) data were obtained from the same crystal at two different temperatures.

**Figure 7:**
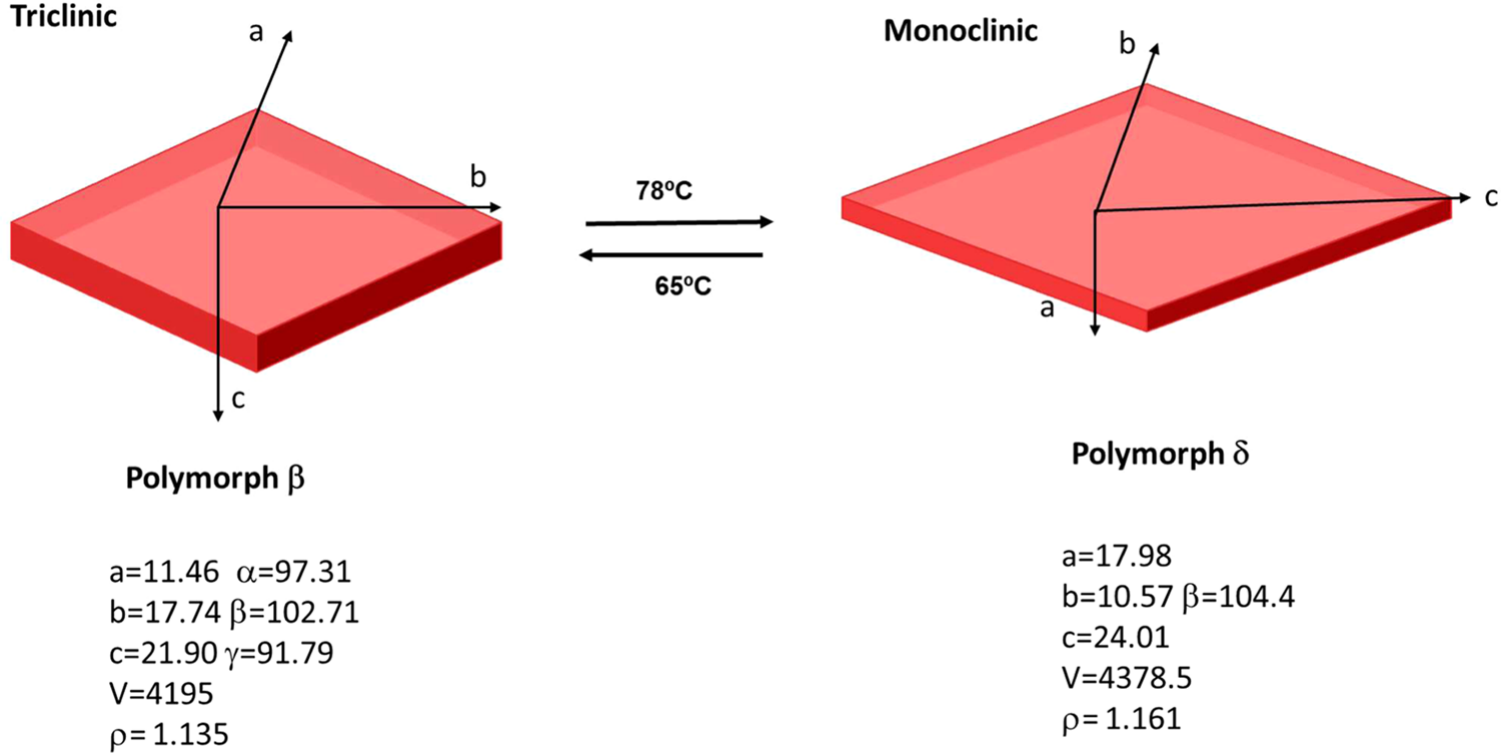
Schematic representation of the overall crystal parameters changes upon the transition between polymorphs **β** and **δ**.

Crystal structure determination indicates that the polymorphic transformation is characterized by substantial changes in the unit cell dimensions (Figure 7). There is an overall expansion of 2.2% in its unit volume, along with an exchange in the magnitudes of the three unit cell axes. Particularly, the *a*-axis and *b*-axis, the *b*-axis and *c*-axis, and the *c*-axis and *a*-axis are interchanged when transitioning from polymorph **β** to **δ**. Upon heating above the temperature of the phase transition, the crystal exhibits a giant positive thermal expansion (35%) along the *b*-axis (that increases from 17.74 to 24.01 Å) and negative thermal expansion along the other two axes (the *a*-axis contracts from 11.46 to 10.57 Å, while the *c-*axis suffers an extraordinarily large shrinkage of 9.6% going from 21.90 to 17.98 Å). Slight differences in the unit cell angles can also be encountered during the polymorphic transformation. The dramatic 33% expansion of a rhombus-shaped single crystal along its diagonal, as depicted in Figure 2, strongly correlates with the crystallographic *b*-axis expansion. On the other hand, the significant shrinkage of the *c*-axis of polymorph **δ**, coinciding with the crystal’s width, provides an explanation for the thermosalient behavior observed when the crystal returns to its initial shape.

A close comparison between the two independent molecules in the asymmetric unit in polymorph **β** and the unique molecule in polymorph **δ** reveals that while there are evident differences in the rotation angles among the five connected aromatic units of **PT-BTD** (see Figures 5a, S14, and S15), there is no alteration in the rotation direction when comparing the molecules across the two polymorphs. Differences are more notable when comparing the peripheral alkyl chains. In polymorph **β**, the alkyl chains are in an all-trans zigzag conformation, and in one of the two independent molecules, they extend along the long axis of the molecule, while in the other molecule, they are highly contorted and oriented in opposing directions. In polymorph **δ**, the torsion angles of the alkyl chains are intermediate between those found in the two conformers of the preceding low-temperature phase and highly affected by the disorder.

**Figure 8:**
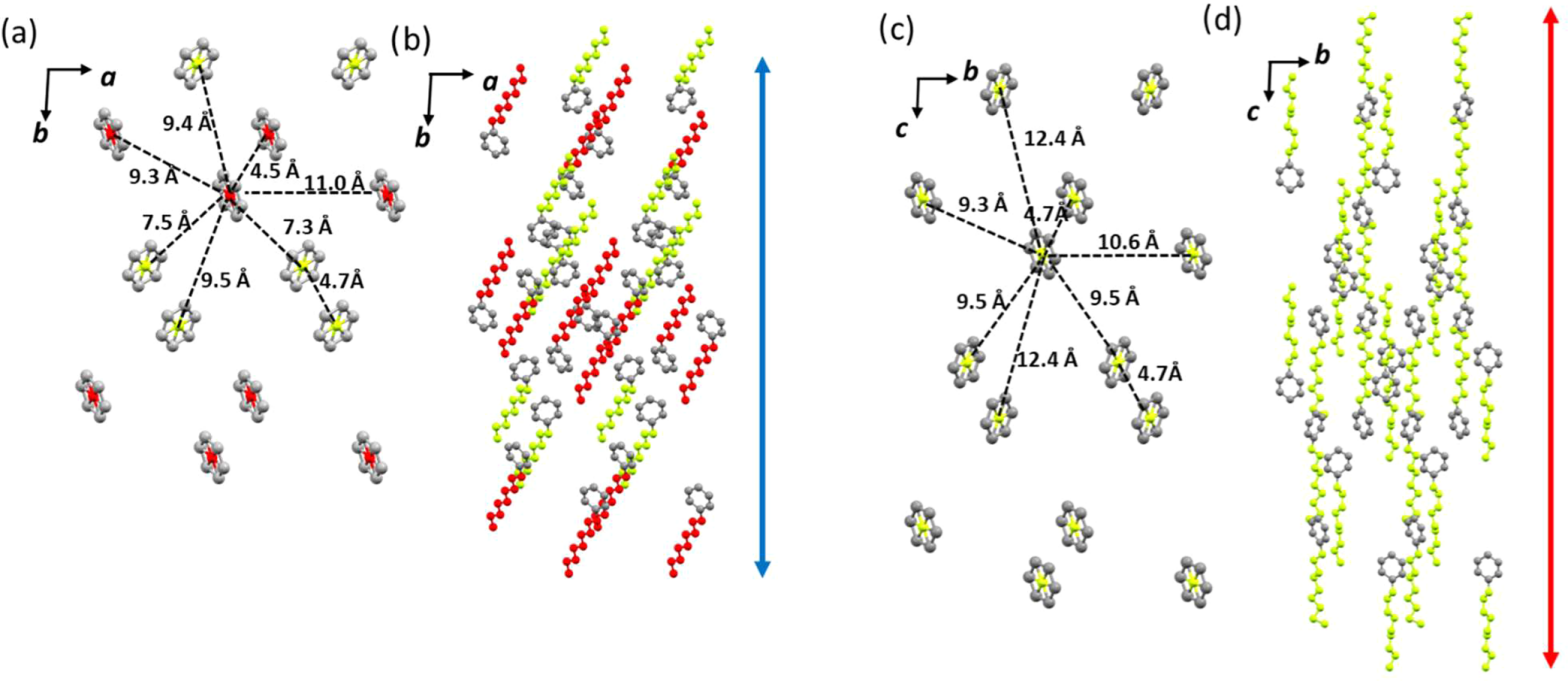
Comparison of the centroids’ positions for the six-membered rings of the central BTD unit (a, c) and the peripheral chains (b, d) in polymorphs **β** and **δ**, viewed along the *c-* and *a-*axes, respectively. The elements of the two different molecules in the asymmetric unit in polymorph **β** are depicted in different colors. The red/blue arrows illustrate the expansion/shrinkage of the *b*/c-axis when heating/cooling the crystals polymorphs **β** and **δ**, respectively.

A comparison of the crystallographic packing along the three crystallographic axes allows us to visualize the expansion/contraction effects that take place in the crystals as a result of these subtle conformational changes after the thermal treatment (see the Supporting Information). Figure 8a,8c illustrates the magnitude of the translational movements suffered by these molecules during the phase transformation. For simplicity, we have only represented the arrangement of the six-membered ring of the central BTD unit viewed perpendicular to the *ab* plane in polymorph **β** (Figure 8a) and to the equivalent *bc* plane in polymorph **δ** (Figure 8c), offering a top-down view of the rhombus-shaped crystal packing. Comparing these figures reveals that most of the molecules become separated up to 3 Å from their neighboring molecules. While these molecular gliding events are significant, they alone cannot account for the impressive crystal expansion observed. Figure 8b,d represents the conformational changes in the peripheral phenyl nonyl moieties of the two polymorphs viewed along the above directions, which, as can be observed, also make a substantial contribution to the overall phenomenon.

**Figure 9:**
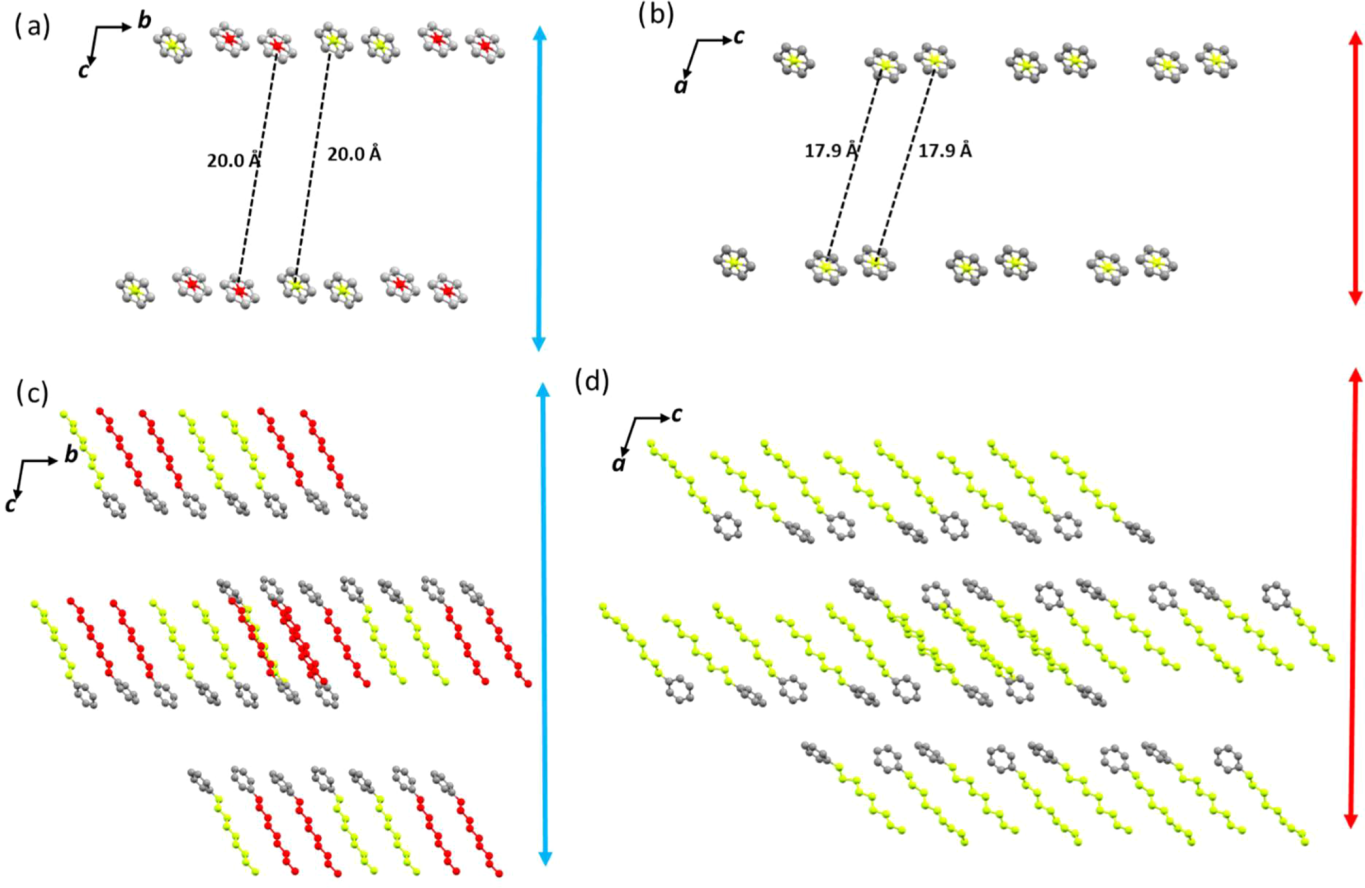
Comparison of the centroids’ positions for the six-membered rings of the central BTD unit (a, c) and the peripheral chains (b, d) in polymorphs **β** and **δ** viewed along the *a*- and *b*-axes, respectively. The elements of the two different molecules in the asymmetric unit in polymorph **β** are illustrated in different colors. The red/blue arrows illustrate the shrinkage/expansion of the *c*/a-axis when heating/cooling the crystals.

Figure 9 illustrates similar comparisons in a view perpendicular to the *bc* plane in polymorph **β** and the equivalent *ac* plane in polymorph **δ**, which show the layered arrangement and provide a lateral view of the packing of the rhombus-shaped crystal. In this case, we can observe a shortening in the distances between the depicted ring of more than 2 Å, accompanied by an inclination of the alkyl chains, which translates into a clear shortening of the interlayer distances emphasizing the cooperation movements and conformational changes in the alkyl chains to explain the overall expansion of the crystal.

### Role of Intermolecular Interactions

**Figure 10:**
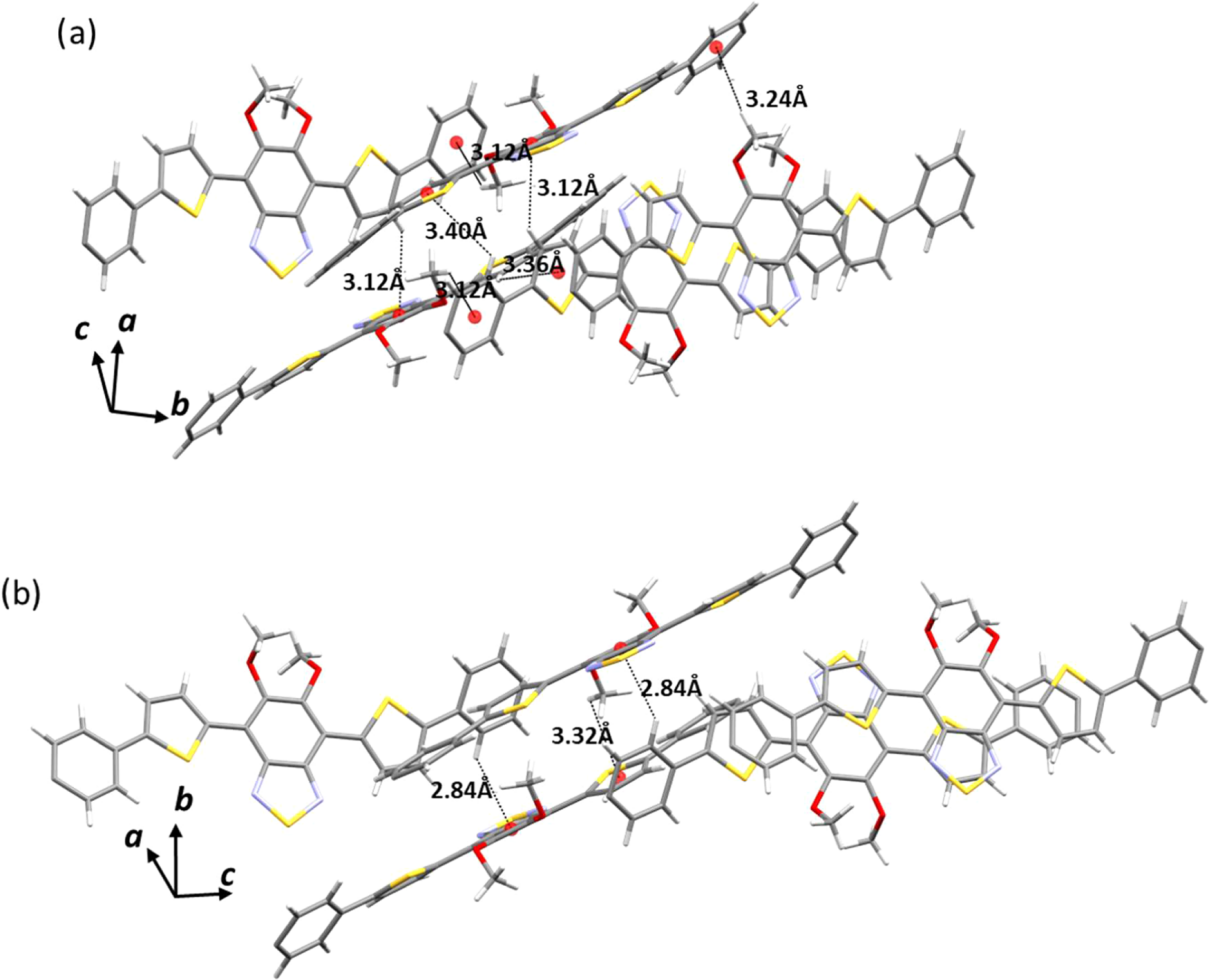
Illustration of how the interaction involving the aromatic section of the molecules changes when transitioning from (a) polymorphs **β** and (b) **δ**. The alkyl chains have been removed for clarity.

In order to understand the distinct thermal behaviors of polymorphs **α** and **β**, we compared their different crystal packing. Interestingly, although molecules in both structures organize into layers, in polymorph **β**, the repeating unit within the layer consists of individual, nonconnected structural units, which are strongly interdigitated with the long alkyl chains. In fact, an analysis of the close contacts between the aromatic units in polymorph **β** reveals only a few CH–π and –OCH_3_–π interactions. It is important to note that although the hydrogen atoms were introduced based on geometry, the distances between carbon atoms confirm the existence of these interactions. These interactions are weak enough to guide the assembly but are easily broken and restored. Interestingly, after thermal transformation into polymorph **δ**, similar interactions can be observed, albeit involving different aromatic rings. Figure 10 illustrates how the interaction involving the aromatic section of the molecules changes as a result of the structural and spatial arrangement. Apparently, such a highly compact yet loosely bound supramolecular structure is highly favorable to accommodate the displacement of the molecules in the crystal packing, resulting in interesting thermoelastic behavior.

**Figure 11:**
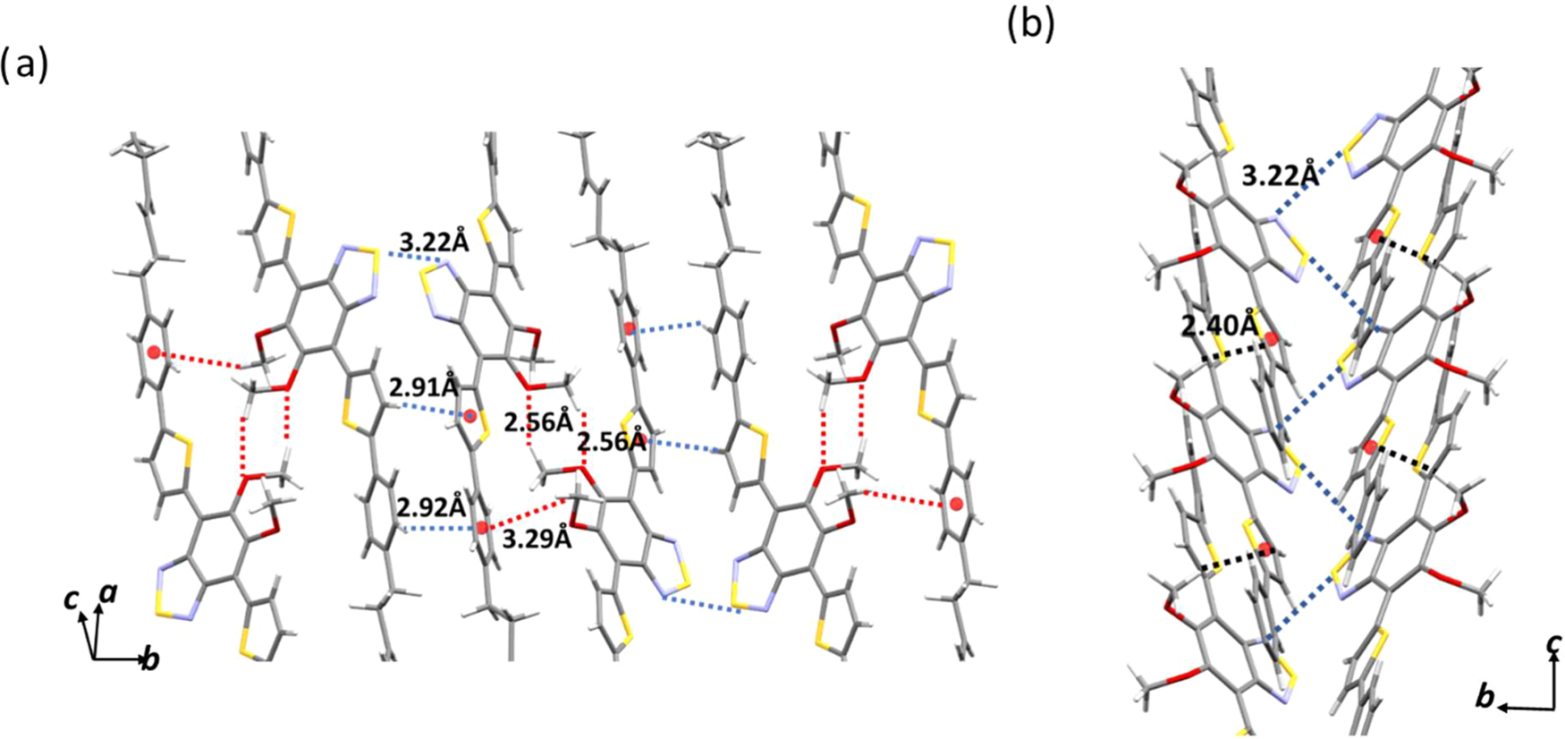
(a) Illustration of the interactions giving rise to the formation of the layers: CH–π and N–S interactions connecting the dimers (in blue) and methoxy–methoxy contacts and OCH_3_–π interactions (in red) between adjacent dimers. (b) Network of N–S interactions (blue) and OCH_3_–π interactions (black) that held together the layers in polymorph **α** (alkyl chains have been omitted for the sake of clarity).

In contrast, in polymorph **α**, the repeating units within each layer form pairs of molecules that are connected by N–S interactions. Layers emerge as a result of a dense interplay of molecular contacts (Figure 11). The molecules assemble into dimers, positioning their BTD units in opposition. These dimers are bound through cooperative CH-π and N–S interactions. Adjacent dimers alternate interacting through methoxy–methoxy contacts and OCH_3_–π interactions. These interactions extend along the *b* direction, forming the layers. The dimers are further organized into stacks, linked together by additional N–S and OCH_3_–π interactions (Figure 11b), contributing to create a robust supramolecular framework, which is not easy to break under the influence of external stimuli.

## Conclusions

In conclusion, we present **PT-BTD**, a novel rod-shaped molecule characterized by a linear arrangement of five rings. The molecule features a centrally positioned dimethoxybenzothiadiazole ring symmetrically functionalized with two thiophene rings. Additionally, each thiophene is linked to a phenyl ring flanked by two flexible nonyl chains. This flexible structure translates to rich polymorphism.

Crystallization experiments involving the diffusion of MeOH vapors into a THF solution of **PT-BTD** resulted in the formation of two different polymorphs (polymorphs **α** and **β**) depending on the duration of the ongoing diffusion process. Under thermal stimulation, polymorph **β** exhibits a remarkable thermosalient behavior, suffering a drastic anisotropic crystal elongation (33% in length of one of its dimensions). Conversely, heating polymorph **α** does not show any visible shape transformation.

A comparison of the crystal packing of the two polymorphs shows that although both form a layer arrangement, there are important differences in terms of interactions among the constituting molecules. While in polymorph **α** layers emerge as a result of a dense interplay of cooperative CH–π, N–S, OCH_3_–OCH_3_, and OCH_3_–π interactions, giving rise to a robust supramolecular framework, not easy to break under the influence of external stimuli, in polymorph **β**, the aromatic part of the molecules are intercalated among the alkyl chains, showing only a few CH–π and –OCH_3_–π resulting in a highly compact yet loosely bound supramolecular structure.

Remarkably, the giant thermosalient transformation of polymorph **β** occurs maintaining the crystal integrity throughout the heating cycle, which has enabled us to determine the structure of the new polymorph on the same crystal. Crystal structure analysis demonstrates that at the molecular level, the thermosalient behavior is associated with subtle coordinated conformational changes, including slight rotations of the five interconnected aromatic units and an enhanced dynamism within one peripheral alkyl chain with rising temperature, triggering the displacements of the molecules.

The result of this study underscores the importance of a soft and flexible structural configuration coupled with a highly compact yet loosely bound supramolecular structure in the development of thermoelastic materials. Our findings emphasize the significance of incorporating structural flexibility, rotating molecular groups, and flexible chains into the design of mechanically active molecular materials to achieve tailored mechanical responses.

## Supporting Information Available

Additional experimental details on the synthesis and characterization of **PT-BTD** (PDF)
Video S1 (MP4)
Video S2 (MP4)
Video S3 (MP4)
