# Supplementary material for: Giant Thermosalient Effect in a Molecular Single Crystal: Dynamic Transformations and Mechanistic Insights
Source: J Am Chem Soc. 2024 Sep 24;146(40):27690–700. doi: 10.1021/jacs.4c09222 (PMC11467902; doi:10.1021/jacs.4c09222)
Supplement: Supplementary file 1 — ja4c09222_si_001.pdf [file ja4c09222_si_001.pdf]

Giant Thermosalient effect in a Molecular Single Crystal: Dynamic Transformations and Mechanistic Insights

Mohammad Afsar Uddin[1,], Raúl Martín[1,2], Sergio Gámez-Valenzuela,[3] Marcelo Echeverri,[1] M. Carmen Ruiz Delgado,[3] Enrique Gutierrez[1], Angeles Monge[1], Berta Gómez-Lor[1,]**

[1] Instituto de Ciencia de Materiales de Madrid, CSIC, Cantoblanco, 28049, Madrid, Spain

[2] Faculty of Chemical and Technologies Sciences, University of Castilla-La Mancha.13071 Ciudad Real, Spain.

[3] Department of Physical Chemistry, University of Málaga, Campus de Teatinos s/n, 29071 Málaga, Spain

Content

1. Experimental details 2
1.1. General 2
1.3. Copy of ^{1}H-NMR and ^{13}C-NMR spectra of 6,7 and PT-BTD 7
2. Photophysical characterization 10
3. Powder X-Ray diffraction of bulk polymorph α and β. 12
4. Thermal Analysis 12
5. Characterization of the mesophase 15
6. Crystal stability 15
7. Single crystal X-ray structure determination of Polymorphs α, β and δ 17
8. Comparison of the crystallographic packing of polymorphs δ and β 19
9. Transformation of polymorphs β into α after melting and cooling. 21
10. Thermosalient Effect visualization 21
11. Examples of previously reported thermosalient materials 22
12. Computational details and DFT calculations 23
12.1. Computational details 23
12.2. DFT calculations 24
13. Experimental and theoretical Raman spectra 25
14. References 31

1. Experimental details

1.1. General

All reagents and solvents employed were commercially available and used as received without further purification. NMR spectra were recorded in a Bruker 200 equipment using $CDCl_3$ as solvent.

Powder X-ray diffraction: Powder X-ray diffraction (PXRD) patterns were measured with a Bruker D8 diffractometer, with step size = 0.02° and exposure time = 0.5 s/step.

Thermogravimetric Analysis (TGA): TGA measurements were performed using a TA Instruments Q500 thermobalance equipped with an EGA furnace. Pt and N_2 sample holders were employed as purge gas with a flow rate of 90 mL/min. The samples were heated from room temperature to 980°C.

Optical Microscopy: The images of the crystals and the optical textures of the mesophases were registered with a Nikon polarizing microscope Eclipse LV 100N POL

equipped with a Linkam hot-stage and Linkam LINKSYS32 central processor and microphotographs were taken with a Nikon DP12 digital camera.

Differential Scanning Calorimetry (DSC): DSC curves were recorded on a TA Instruments Discovery DSC calorimeter, utilizing standard TzeroTM sample holders. The purge gas used was N_2 at a flow rate of 50 mL/min, and a heating ramp of 10ºC/min from -50 °C to 200 °C was applied.

Raman spectra: Raman spectra were recorded by using the 1x1 camera of a Bruker Senterra dispersive Raman microscope equipped with a CCD camera operating at -50ºC, a confocal microscope with a x40 objective, a neon lamp and a Nd:YAG laser working at λ=785 nm. This set up has a spectral resolution of 3-5 cm^{-1}. The power of the laser beam was kept at a level of 25 mW in all cases. The final spectrum is calculated as the average between 20 spectra of 10 seconds each to optimize the signal-to-noise ratio. The temperature of the sample was controlled by a Linkam temperature stage (BCS196).

FT-IR spectra: FT-IR spectra were recorded with a Golden Gate Single Reflection Diamond ATR System (Graseby Specac) fitted into a Bruker Vertex 70 FT-IR spectrometer. The infrared spectra were collected with a standard spectral resolution of 4 cm^{-1}. The final spectrum is calculated as the average between 5 spectra of 64 scans each with the aim to obtain a high signal to noise ratio.

Linear Spectroscopy: UV-Visible studies were carried out on a Varian Cary 5000 spectrophotometer. Fluorescence spectra were recorded on an Edinburgh Instruments FLS920 fluorometer.

Fluorescence quantum yield: The absolute quantum yield was registered in an Edinburgh Instruments FLS920 fluorometer, equipped with an integrating sphere, using a 450W Xe lamp as excitation source and the supplied blanking plug as blank sample. Lifetime measurements were carried out using an EPL-475 picosecond pulsed laser as excitation source and a high-speed F-G05 detector.

2. Synthesis and characterization of PT-BTD

Compound 4,7-Dibromo-5,6-dimethoxy2,1,3-benzothiadiazole (**5**) was synthesized following the reported procedure shown in Scheme 1.[1, 2] Compounds **6**, **7** and **PT-BTD** were synthesized by following Scheme 2.

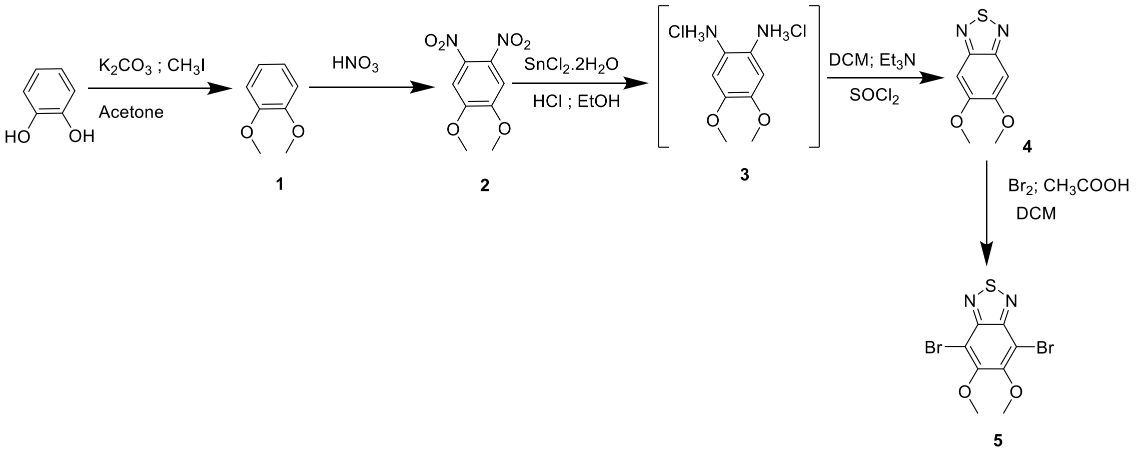

Scheme 1. Synthesis of 4,7-dibromo-5,6-dimethoxybenzo[c][1,2,5]thiadiazole (5)

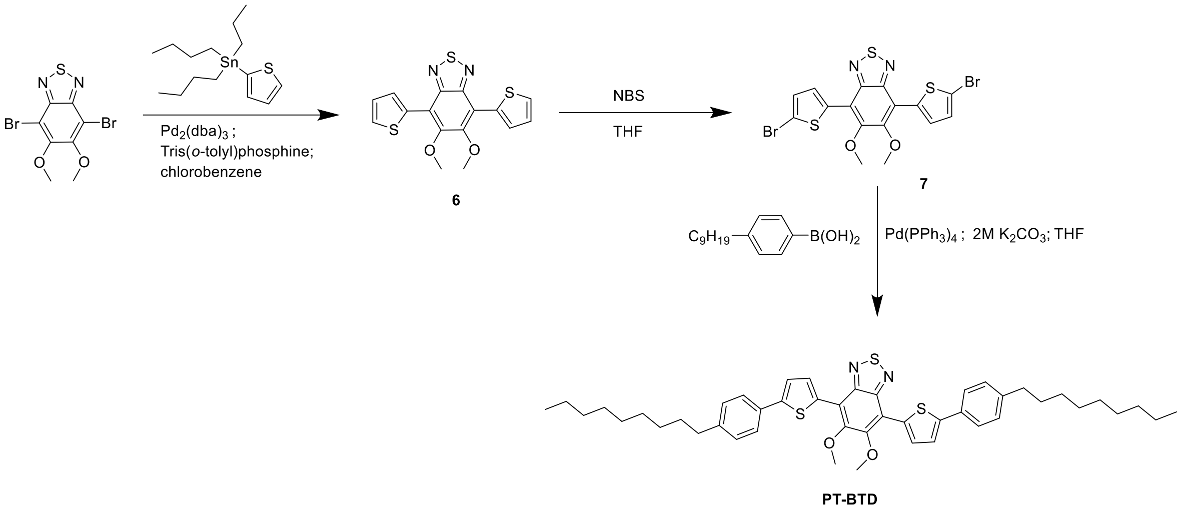

Scheme 2. Synthesis of **PT-BTD**

5,6-Dimethoxy-4,7-di(thiophen-2-yl)benzo[c][1,2,5] thiadiazole (6)

2-(Tributylstannyl)thiophene (2.90 g, 7.77 mmol), 4,7dibromo-5,6-dimethoxybenzo[c][1,2,5]thiadiazole (1.10 g, 3.11 mmol), tris(dibenzylideneacetone)dipalladium(0) (4 mol%), tri(o-tolyl)phosphine (8 mol%) and 3 mL chlorobenzene were added in a 5 mL microwave vial. The stille coupling reaction

mixture was heated at 120 °C 60 minutes in a microwave reactor. The solvent was removed under reduced pressure and the crude product was purified via silica gel column chromatography, eluting with Hexene/dichloromethane (9:1, v:v) to afford 5,6-dimethoxy-4,7-di(thiophen-2-yl)benzo[c][1,2,5] thiadiazole as an orange solid (1.00 g, 89 % yield). ^{1}H NMR (300 MHz, CDCl3, 25 ºC, ppm) 8.61 (m, 2H); 7.50 (m, 2H) 7.24 (m, 2H). 4.04 (s, 6H). ^{13}C NMR (75 MHz, CDCl3, 25 ºC, ppm) 152.49, 151.01, 134.22, 130.91, 127.79, 127.17, 117.48, 60.85.

4,7-Bis(5-bromothiophen-2-yl)-5,6-dimethoxybenzo[c][1,2,5]thiadiazole (7)

NBS (1.23 g, 6.91 mmol) was added to a solution of 5,6-dimethoxy-4,7di(thiophen-2-yl) benzo[c][1,2,5]thiadiazole (1.00 g, 2.77 mmol) in chloroform (100 mL). The mixture was stirred at room temperature for 48 hours in the dark. The chloroform layer was washed with water (3 x 100 mL). The combined organic phase was dried with $MgSO_4$ and the solvent was removed under reduced pressure. The crude product was purified via silica gel column chromatography, eluting with petroleum Hexane/dichloromethane (4:1, v:v) to afford 4,7-bis(5-bromothiophen-2-yl)-5,6dimethoxybenzo[c]1,2,5] thiadiazole as an orange solid (1.15 g, 80 % yield). ^{1}H NMR (300 MHz, CDCl3, 25 ºC, ppm) 8.48 (m, 2H,). 7.17 (m, 2H), 4.04 (s, 6H); ^{13}C NMR (300 MHz, CDCl3, 25 ºC, ppm) 151.75, 150.19, 135.53, 131.14, 129.83.

4,7-bis[5-(4-nonylphenyl)-2-thienyl]-5,6-dimethoxy-2,1,3-benzothiadiazole (PT-BTD)

A solution of 4,7-Bis(5-bromothiophen-2-yl)-5,6-dimethoxybenzo[c][1,2,5]thiadiazole (7) (156 mg, 0.30 mmol), $Pd(PPh_3)_4$ (58.8 mg, 0.050 mmol), 4-nonylphenylboronic acid (148.9 mg, 0.60 mmol) in a mixture of 2 M aqueous K_2CO_3 (0.3 mL) and dry THF (2.0 mL) was degassed and irradiated with a microwave irradiator at 120 °C (80 W) for 120 min. After cooling to room temperature, the reaction mixture was diluted with CH_2Cl_2,

washed with water, and dried over $MgSO_4$ anhydrous. Then, the solvent was evaporated and the residue was purified by column chromatography with hexane/CH_2Cl_2 (3:1) to give 4,7-bis[5-(4-nonylphenyl)-2-thienyl]-5,6-dimethoxy-2,1,3-benzothiadiazole (**PT-BTD**) as an orange solid (150 mg, 65% yield). ^{1}H NMR (300 MHz, CDCl3, 25 ºC, ppm) 8.68 (m, 2H); 7.65 (m, 4H), 7.42 (m, 2H), 7.24 (m, 4H). 4.10 (s, 6H), 2.64 (m, 4H), 1.64 (m, 4H), 1.28 (m, 24H), 0.88 (m, 6H); ^{13}C NMR (300 MHz, CDCl3, 25 ºC, ppm) 151.92, 150.71, 146.00, 142.73, 133.02, 131.93, 131.75, 128.94, 125.81, 122.72, 117.05, 60.64, 35.75, 31.89, 31.42, 29.56, 29.53, 29.33, 22.67,14.12.

1.3. Copy of ^{1}H-NMR and ^{13}C-NMR spectra of 6,7 and PT-BTD

^{1}H-NMR and ^{13}C-NMR spectra of compound 6

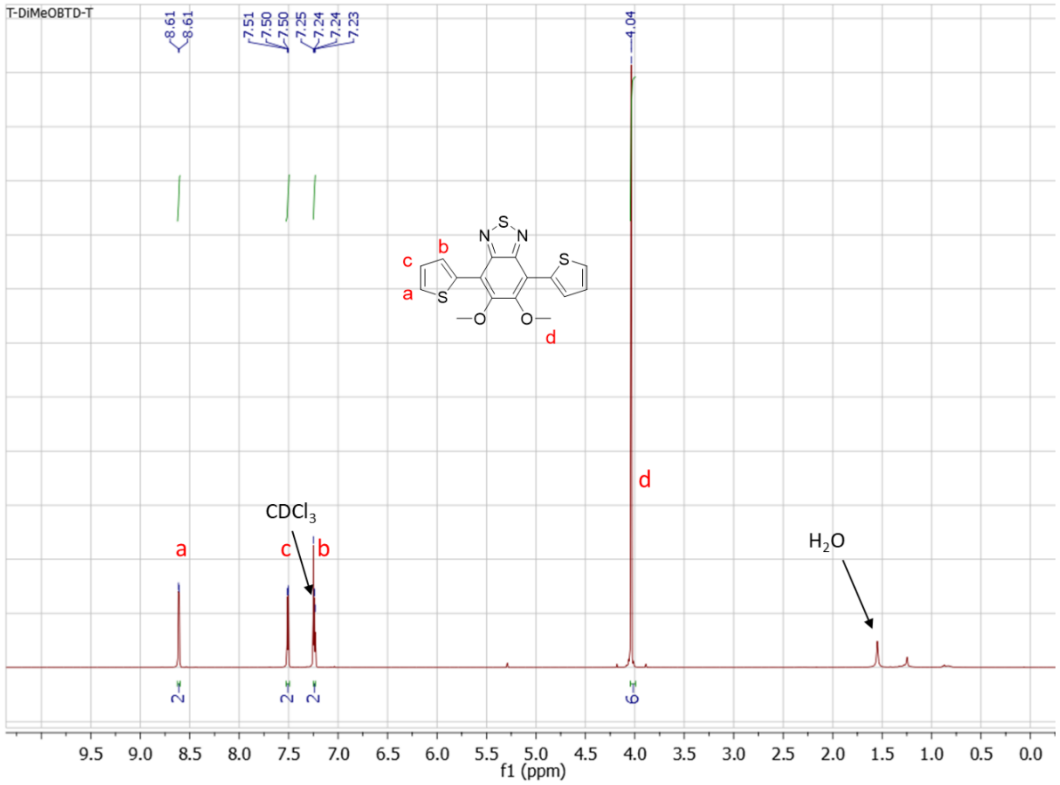

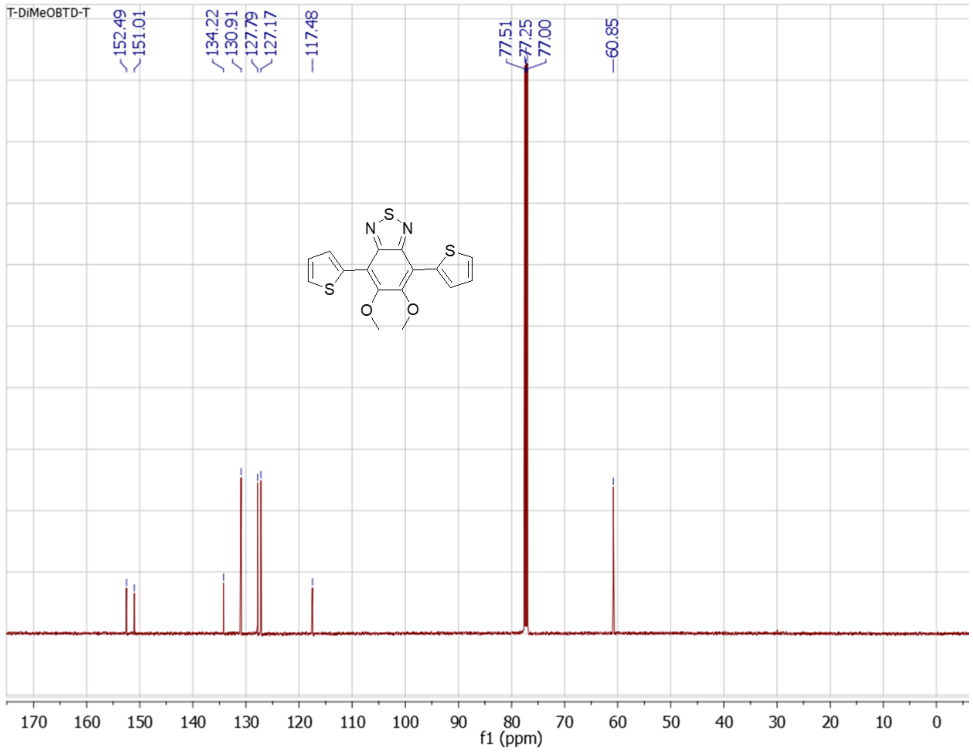

^{1}H-NMR and ^{13}C-NMR spectra of compound 7

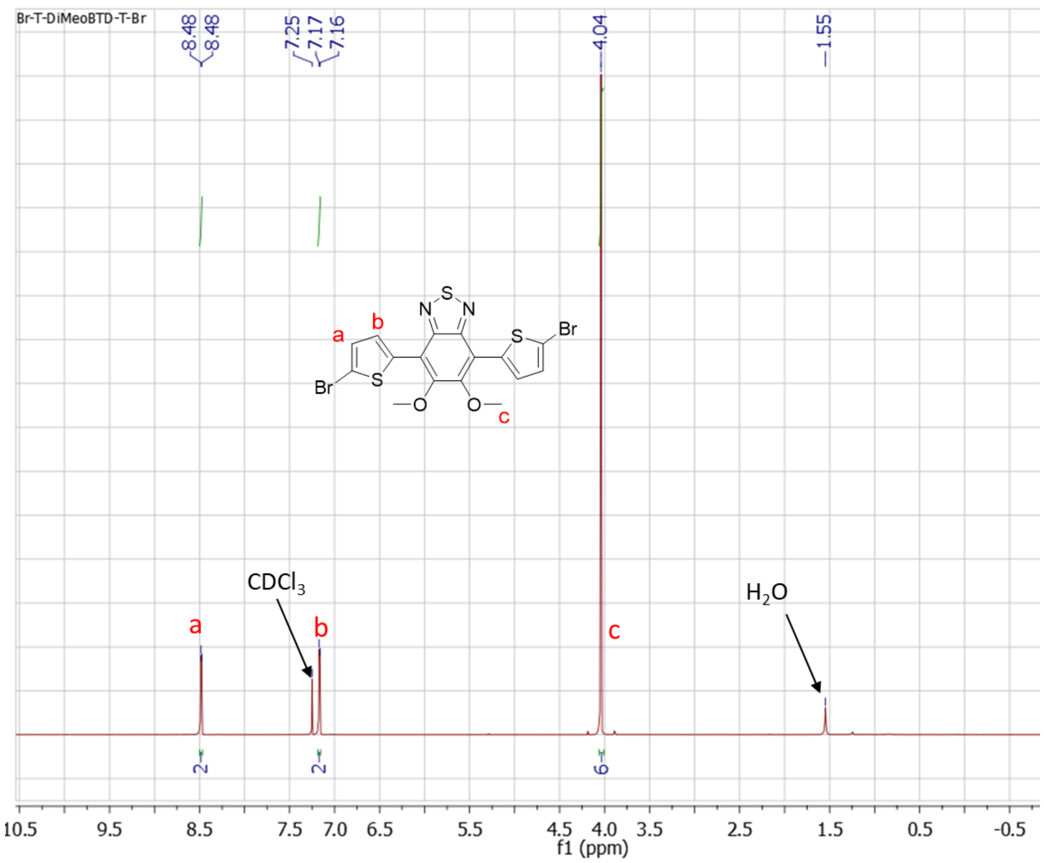

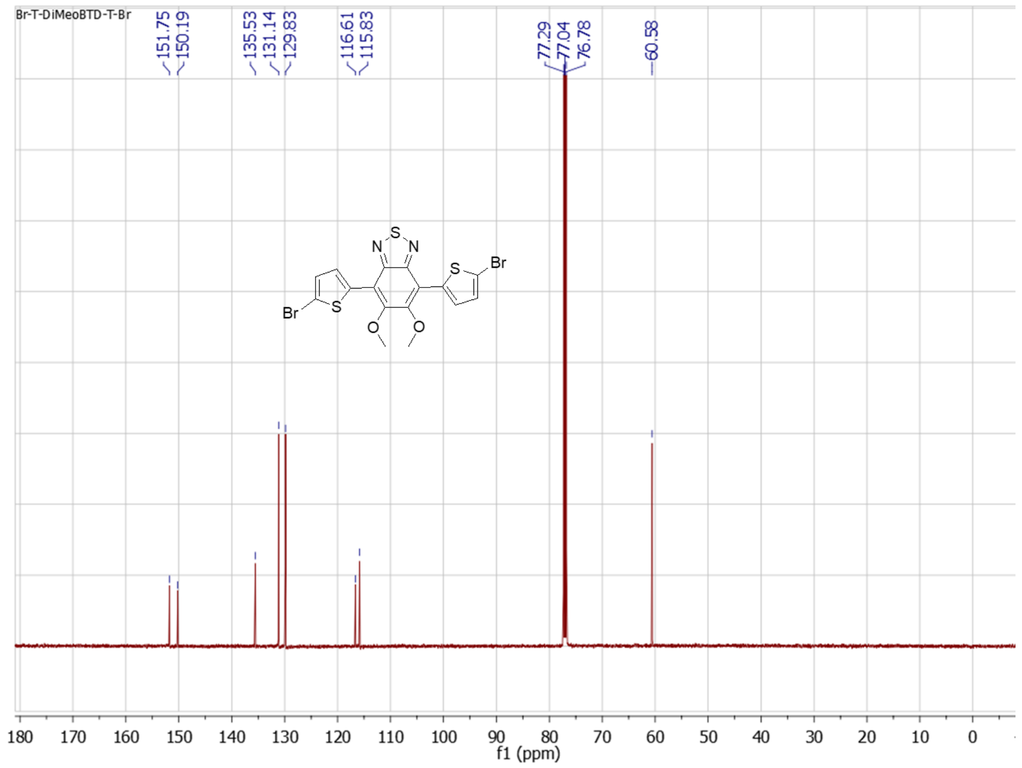

^{1}H-NMR and ^{13}C-NMR spectra of PT-BT

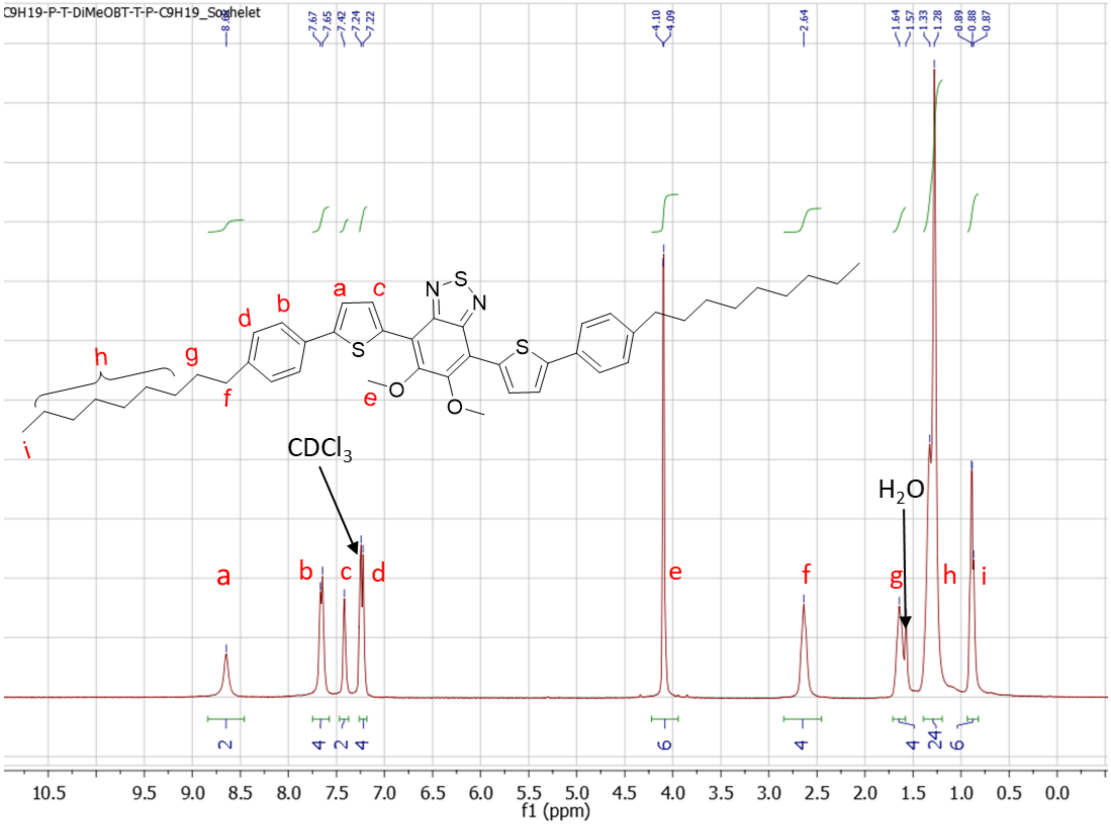
C9H19-P-T-DiMeOBT-T-P-C9H19_Soxhelet
CDCl3
H2O
a
b
c
d
e
f
g
h
i
f1 (ppm)

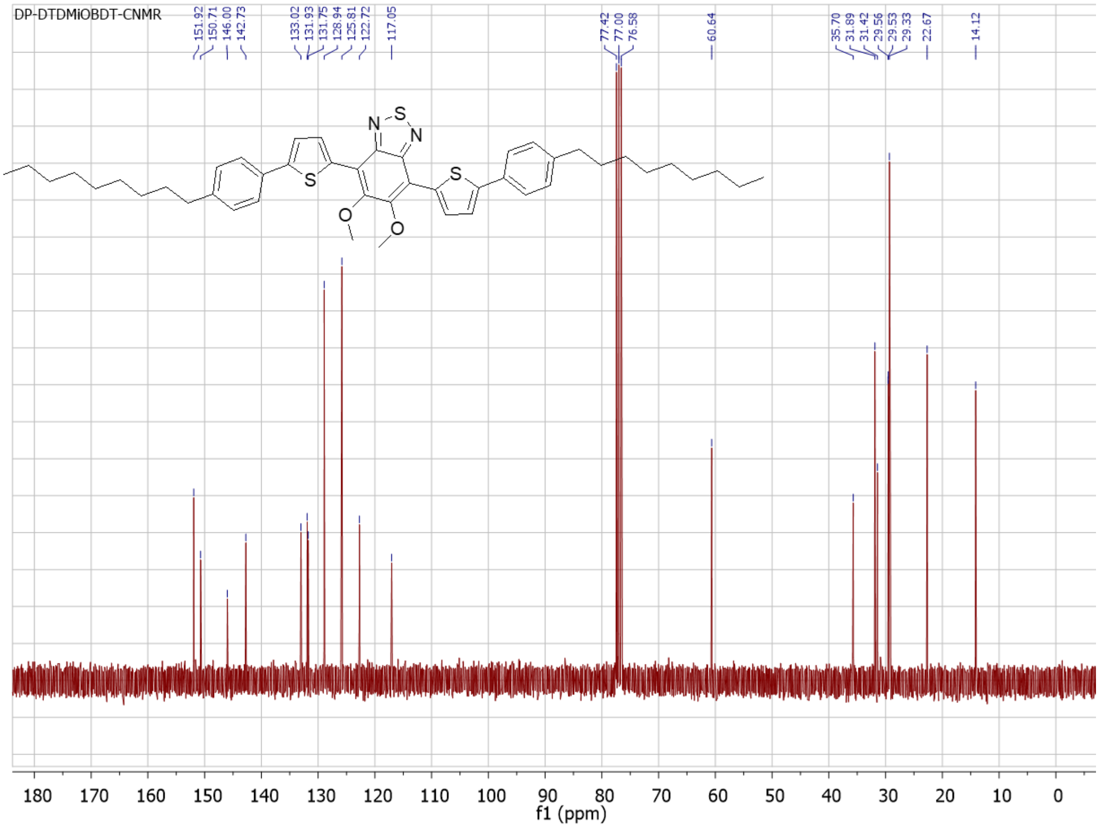
DP-DTDMiOBDT-CNMR
f1 (ppm)

2. Photophysical characterization

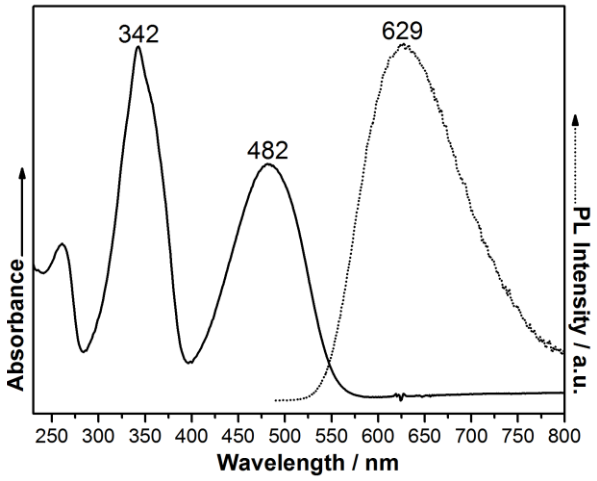

Figure S1. UV−vis absorption (solid line) and fluorescence (dash line) spectra of **PT-BTD** in dichloromethane at a concentration of 10^{-4} M. For emission spectrum, an excitation wavelength of 475 nm has been used.

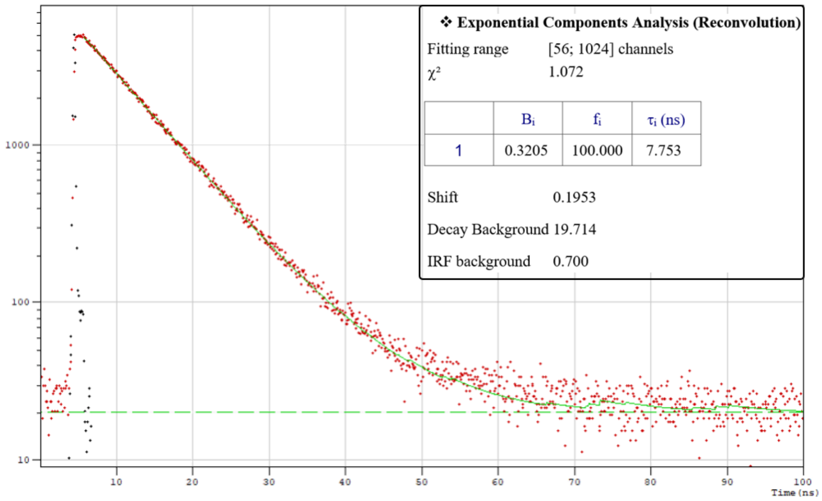

Figure S2. Fluorescence decay of the compound under study solved in dichloromethane. An excitation wavelength of 475 nm has been used.

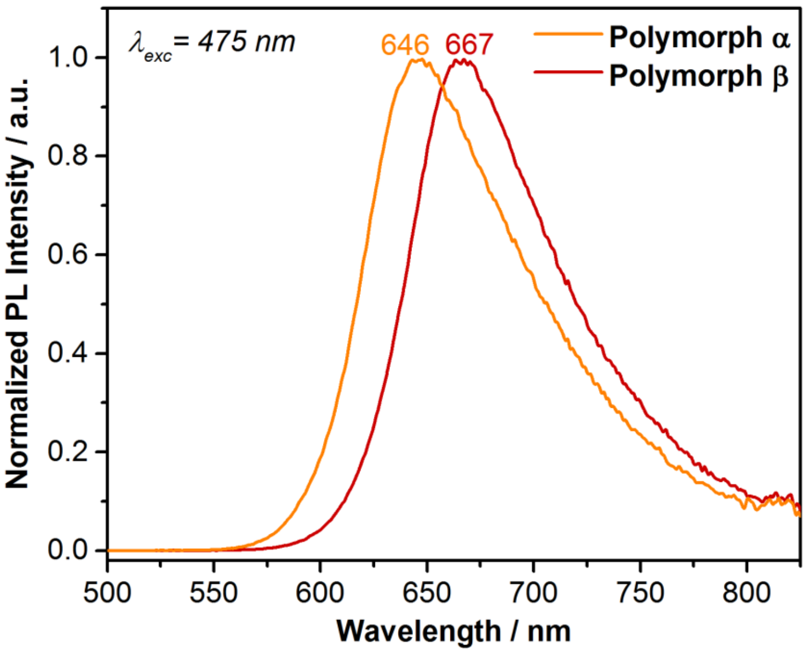

Figure S3. Solid fluorescence spectra of polymorphs α and β of **PT-BTD** when excited at 475 nm.

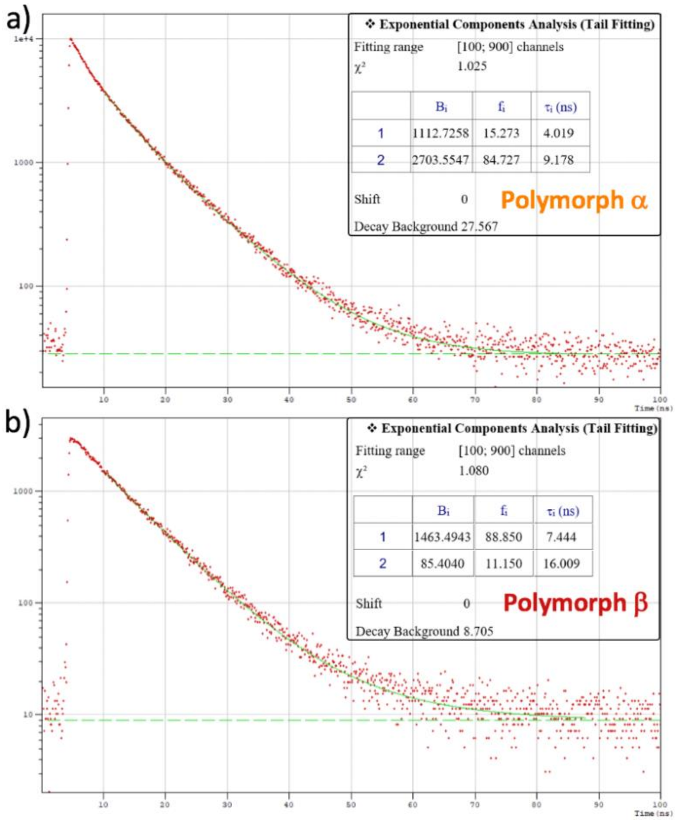

Figure S4. Fluorescence decays of (a) polymorph α and (b) polymorph β with an excitation wavelength of 475 nm. Decays show biexponential character.

3. Powder X-Ray diffraction of bulk polymorph α and β.

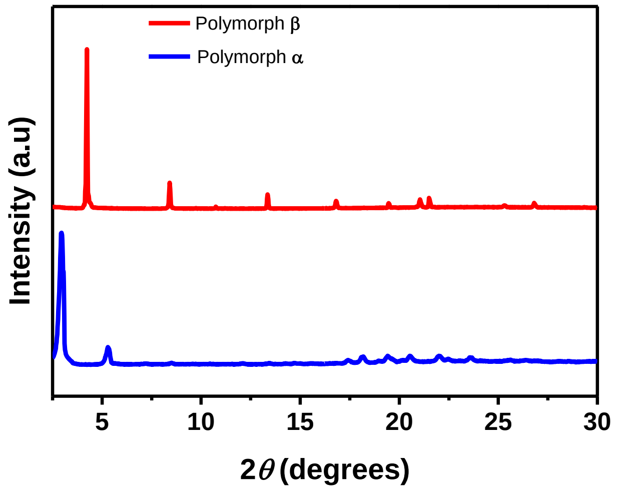

Figure S5. PXRD patterns of the bulk orange thin crystals initially formed by diffusion of non-solvent MeOH vapors into a THF solution of **PTBTD** (polymorph **α**) and of the bulk red rhombus-shaped crystals formed as the THF solution gradually becomes enriched with MeOH (polymorph **β**).

4. Thermal Analysis

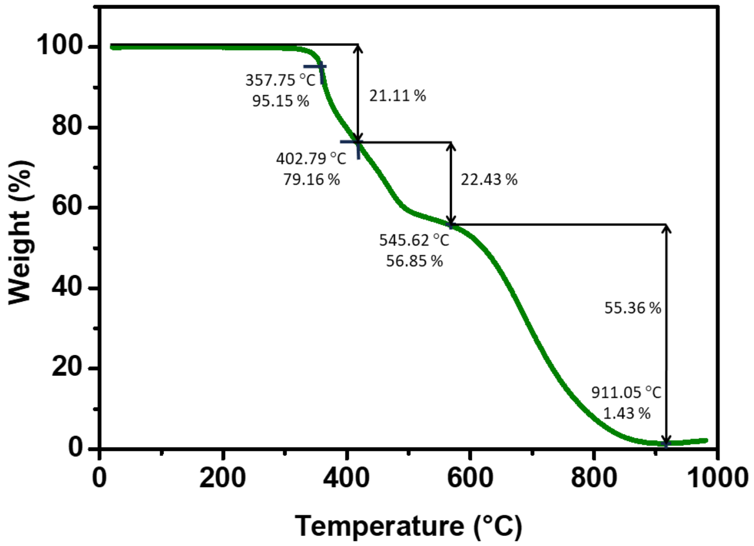

Figure S6. Thermogravimetric analysis (TGA)

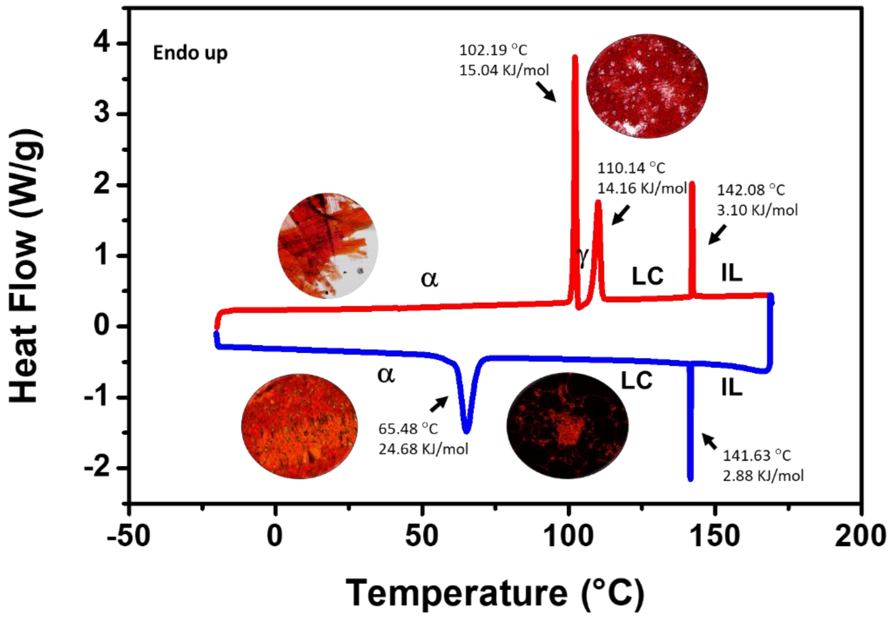

Figure S7. Differential scanning calorimetry of polymorph **α**, showing the textures of the different phases observed under the polarizing optical microscope.

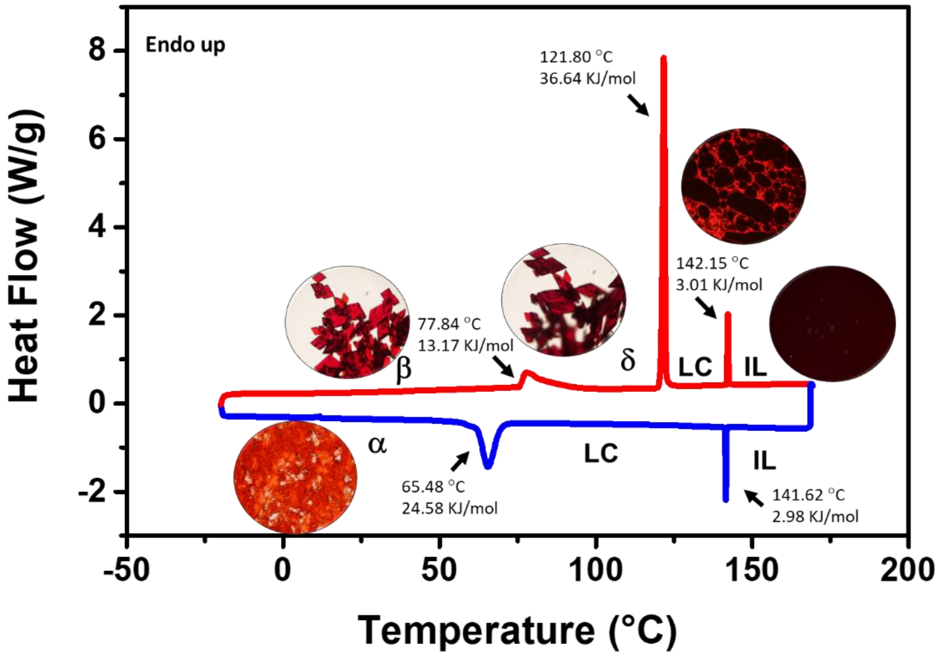

Figure S8. Differential scanning calorimetry of polymorph **β: ** First heating and cooling cycles, showing the textures of the different phases observed under the polarizing optical microscope.

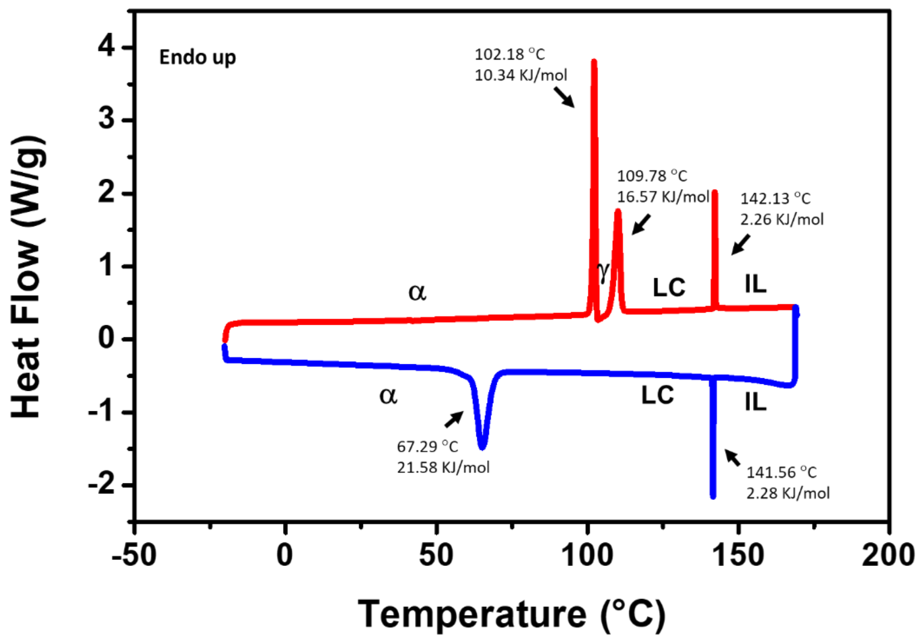

Figure S9. Differential scanning calorimetry of polymorph **β:** Second heating and cooling cycles.

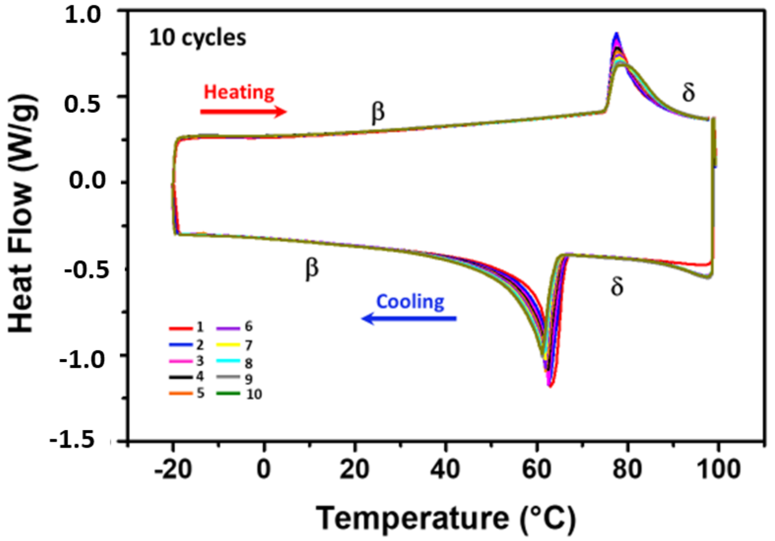

Figure S10. Differential scanning calorimetry of polymorph **β** showing successive cycles in the range of -20 to 100ºC.

5. Characterization of the mesophase

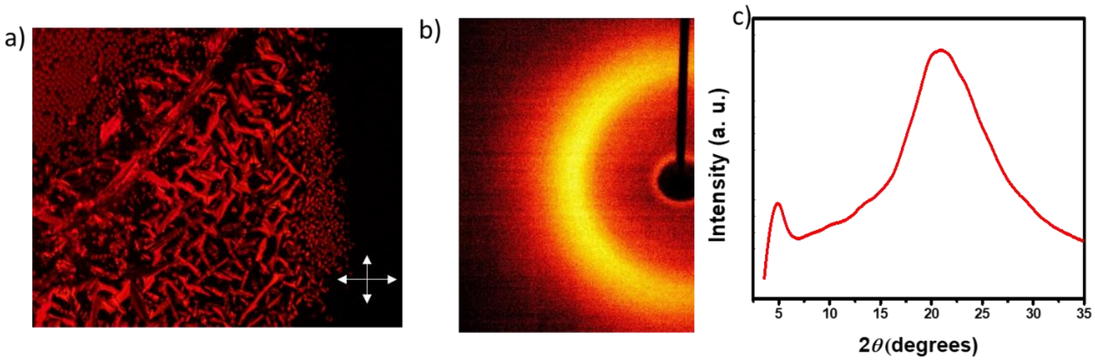

Figure S11. a) Polarizing optical photomicrograph (x20) b) 2D X-ray pattern c) 1D X-ray pattern of the mesophase

The diffractogram of PT-BTD in the mesophase is consistent with a smectic C mesophase. This assignment is supported by the typical broken-fan texture observed in the mesophase through polarized optical microscopy (POM) and by its X-ray diffraction pattern. The diffraction pattern reveals a maximum in the small-angle region, corresponding to the first-order reflection of the smectic layer, and a diffuse halo in the high-angle region, indicative of conformational disorder in the liquid-like chains. The layer spacing was determined to be 18.4 Å by applying Bragg's law to the small-angle maximum. This spacing is significantly shorter than the molecular length (39.9 Å), suggesting strong interdigitation of the alkyl chains and that the molecules are tilted within the layers.

6. Crystal stability

A stability test of the stimuli-responsive polymorph β of **PT-BTD** was performed by soaking crystals in different solvents and at various pH values. We found that, while the crystals remain stable in aqueous acid or basic conditions, and in polar solvents, they dissolve in apolar ones. Additionally, we exposed the crystals to UV light and observed no change in their shape. Regarding the thermal stability, we observed that as long as the temperature is maintained between room 25 and 70 ºC and between 78 and 100 ºC, the shape of the polymorph β and δ, respectively remains unchanged for several hours.

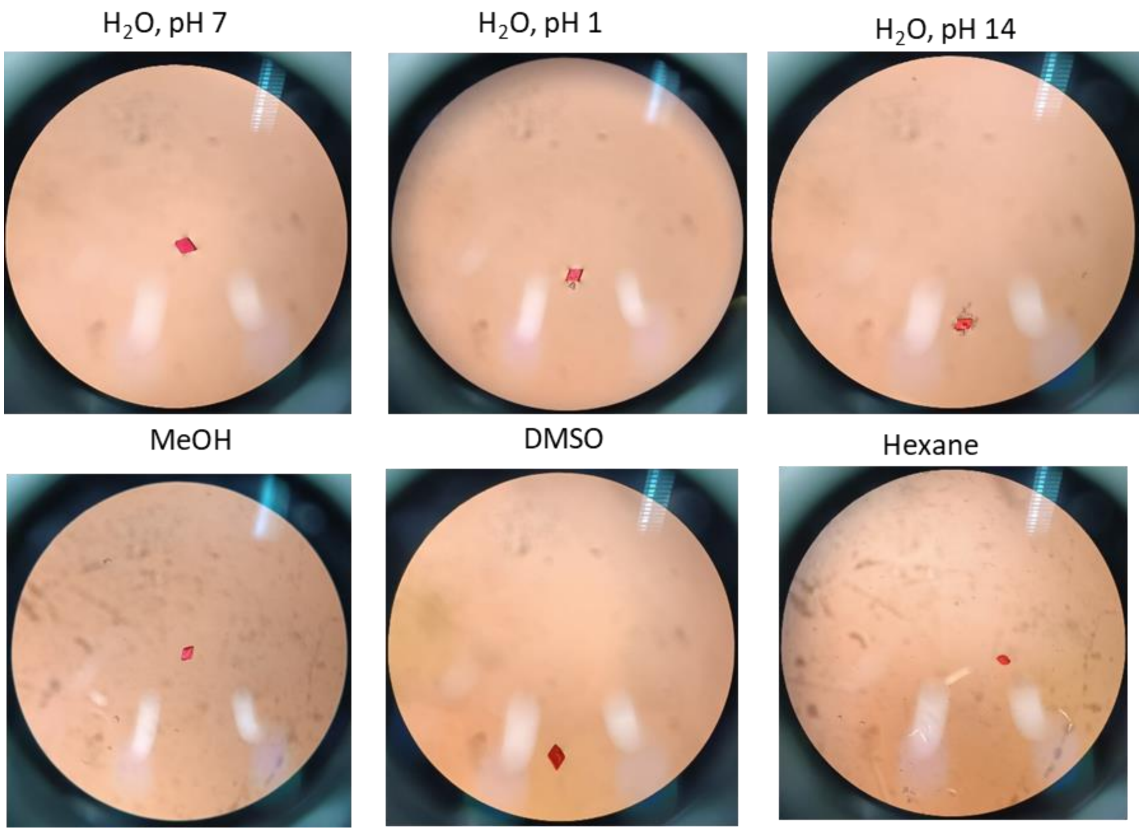

Figure S12. Crystal (polymorph β) stability test in various solvents at room temperature during 48 hours.

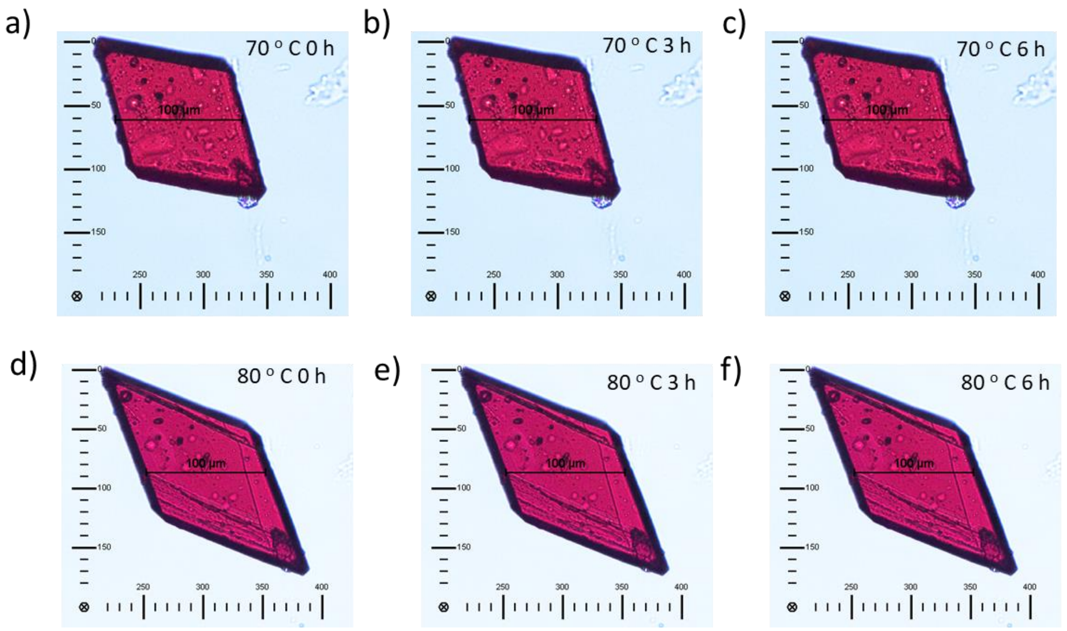

Figure S13. Crystal thermal stability test of polymorph β (a-c) and polymorph δ (d-f).

7. Single crystal X-ray structure determination of Polymorphs α, β and δ

Crystals were selected under a polarizing optical microscope and glued on a glass fiber for a single-crystal X-ray diffraction experiment.

Polymorph α: X-Ray single crystal data of **polymorph α** were collected in a Bruker D8 Venture diffractometer, equipped with a Incoatec Diamond microsource operating at 90 W to generated Cu Kα radiation (1.54178 Å) and a Bruker PHOTON III area detector. Single crystal X-Ray diffraction data were collected exploring over a hemisphere of the reciprocal space in a combination of φ and ω scans

Polymorphs β and δ: A prismatic crystal of **polymorph β** were mounted on a Bruker X8 diffractometer equipped with a Incoatec microsource, operated at 45 w power (50KV, 0.90 mA) to generated Cu Kα radiation (1.54178 Å) and a Bruker PHOTON II area detector. Single crystal X-Ray diffraction data were collected exploring a hemisphere of the reciprocal space at room temperature. After the data collection, the crystal was heated to 363K using a Temperature device (380-110K), observing an increment of crystal size indicating the change to polymorph δ, then a hemisphere of the reciprocal space data was collected keeping the temperature at 363K.

Unit cell dimensions were determined for least-squares fit of reflections with $I > 20\ \sigma$. A semi-empirical absorption and scale correction based on equivalent reflection was carried out. The structures were solved by direct methods. The final cycles of refinement were carried out by full-matrix least-squares analyses with anisotropic thermal parameters of all non-hydrogen atoms. The hydrogen atoms were fixed at their calculated positions using distances and angle constraints. All calculations were performed using APEX3[3] software for data collection and data reduction and SHELXTL[4] and OLEX2[5] to resolve and refine the structure, respectively. CCDC 2313851, CCDC 2313852 and CCDC 2313853 contain the supplementary crystallographic data for polymorphs α, β and δ of **PT-BTD** These data can be obtained free of charge via www.ccdc.cam.ac.uk/data_request/cif, or by emailing data_request@ccdc.cam.ac.uk, or by contacting The Cambridge Crystallographic Data Centre, 12 Union Road, Cambridge CB2 1EZ, UK; fax: +44 1223 336033.

Explanation to CheckCIF A alerts in polymorph α: As all the crystals were polysynthetically twinned, some reflections showed illogical Fo-Fc values, likely due to

the contribution from multiple components. These reflections were omitted, even in this, our best data collection. The “low diffrn_measured_fraction_theta_full value” alert is a consequence of this omission, as well as the intrinsic difficulty in accurately modeling the twinned crystal structure.

<u>Explanation to CheckCIF A alerts in polymorph δ:</u> Due to the significant changes in the crystal dimensions following the high-temperature transition and the subsequent reorganization of the long aliphatic chains, some carbon atoms had to be refined isotropically with high Ueq values. This transition led to increased atomic displacement parameters (Ueq), reflecting the dynamic nature of the aliphatic chains. The calculated H...H inter-chain distances may not be reliable under these conditions.

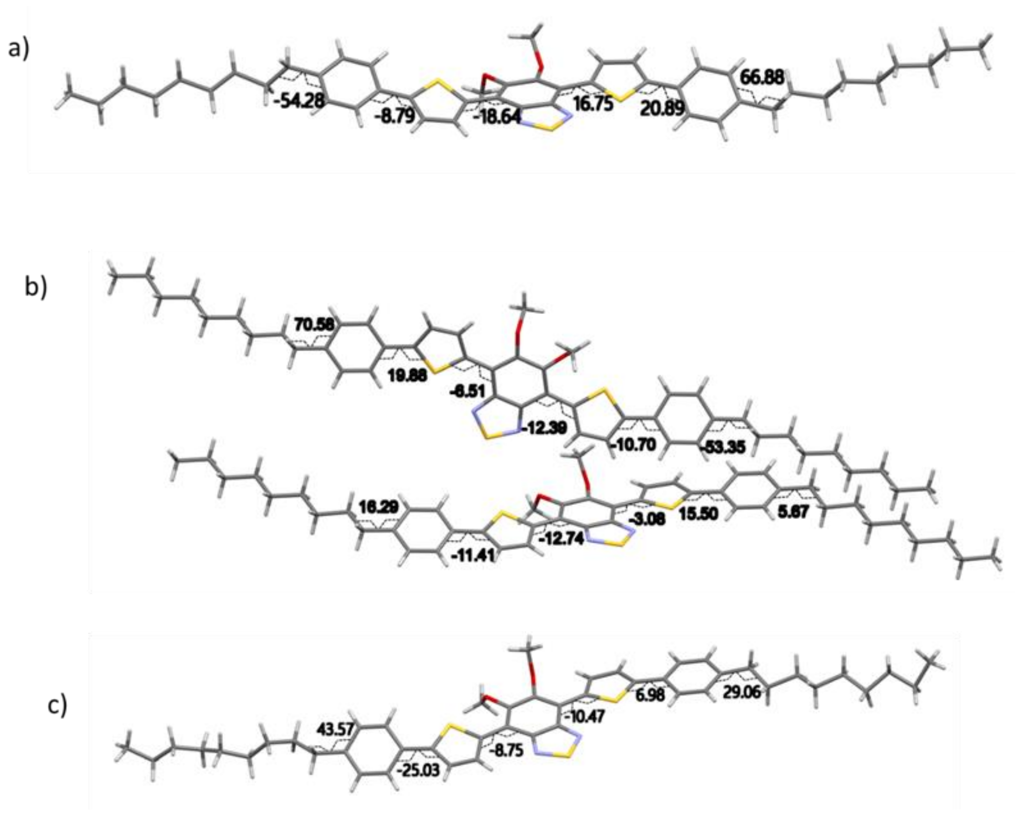

Figure S14: View of the independent molecules of **PT-BTD** in polymorphs (a) α , (b) β and (c) δ, showing variation in the representative angles.

8. Comparison of the crystallographic packing of polymorphs δ and β

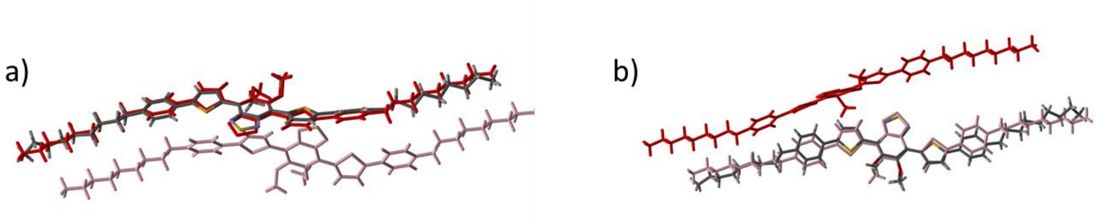

Figure S15. Overlap of the crystallographically unique molecule in polymorph δ with the two different conformers (in red and pink) of polymorph β.

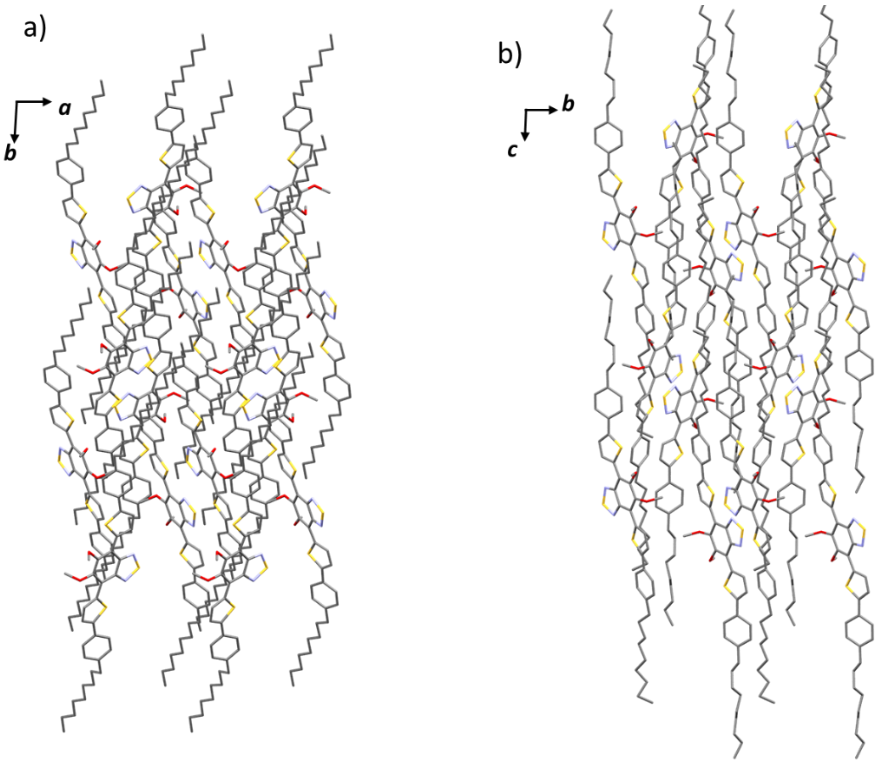

Figure S16. Crystallographic packing of polymorphs β (a) and δ (b) viewed along *c* and *a* axis respectively.

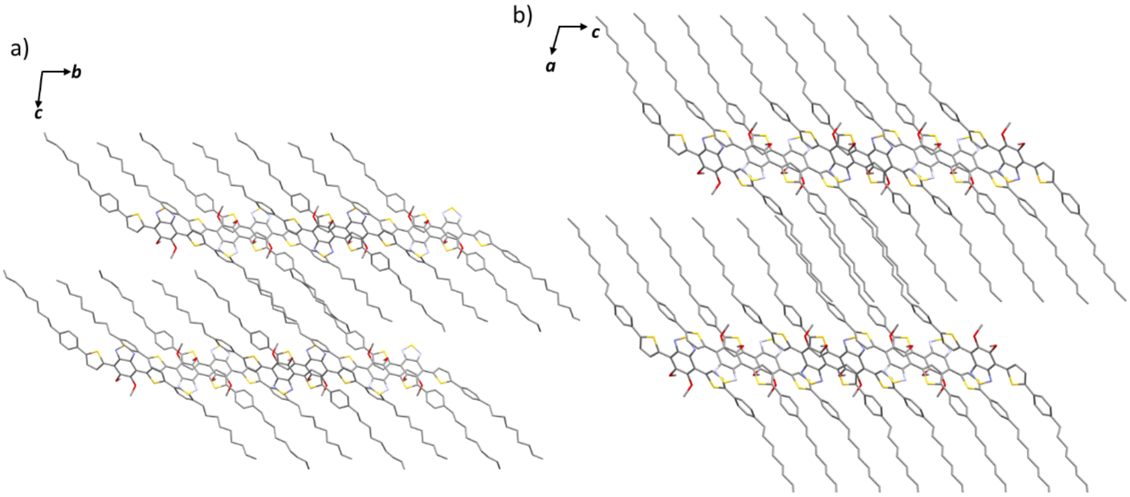

Figure S17. Crystallographic packing of polymorphs β (a) and δ (b) viewed along *a* and *b* axis respectively.

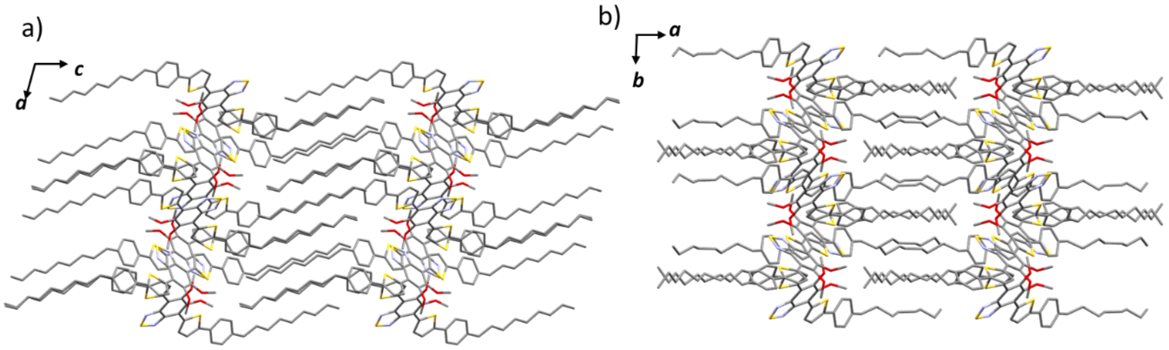

Figure S18. Crystallographic packing of polymorphs β (a) and δ (b) viewed along *b* and *c* axis respectively.

9. Transformation of polymorphs β into α after melting and cooling.

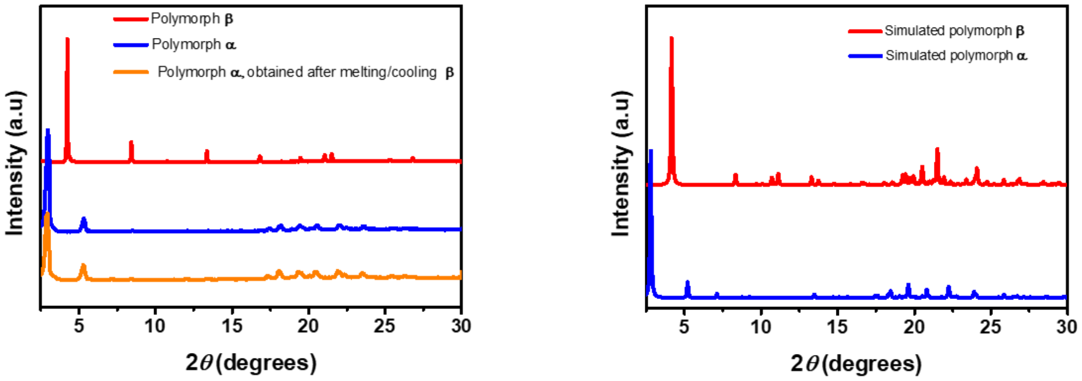

Figure S19. a) Comparison of the PXRD pattern of polymorph α growth by slow evaporation and the phase obtained after melting and subsequent cooling to room temperature the polymorph β. b) PXRD pattern of polymorphs α and β simulated from single crystal X-ray data.

10. Thermosalient Effect visualization

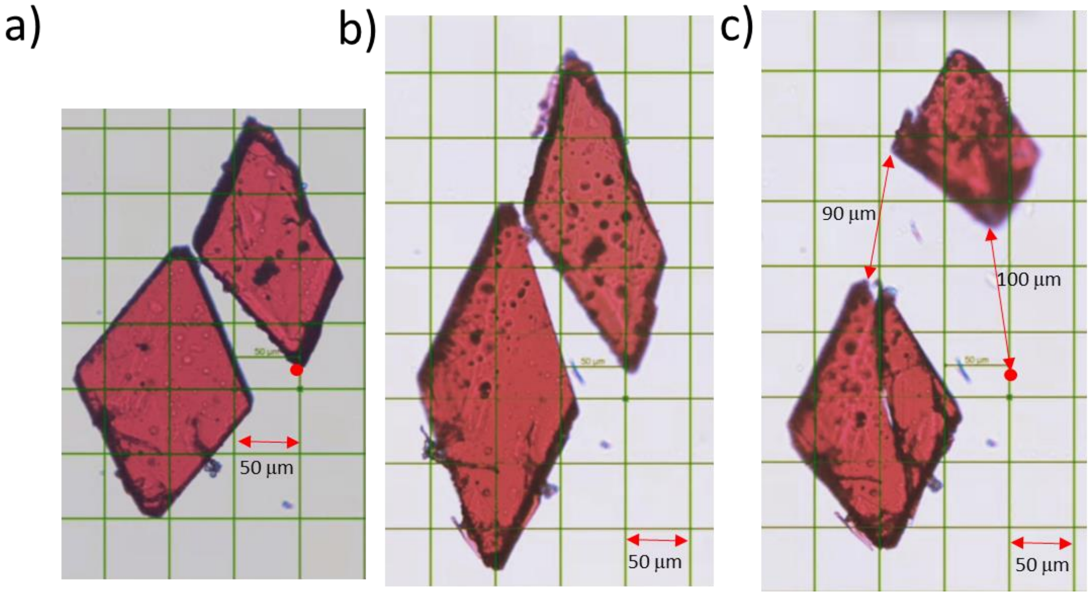

Figure S20. Crystal displacement measurements upon heating/cooling polymorph β/δ. Images are obtained starting on polymorph β at a) room temperature b) 80 ºC and c) cooling back to room temperature.

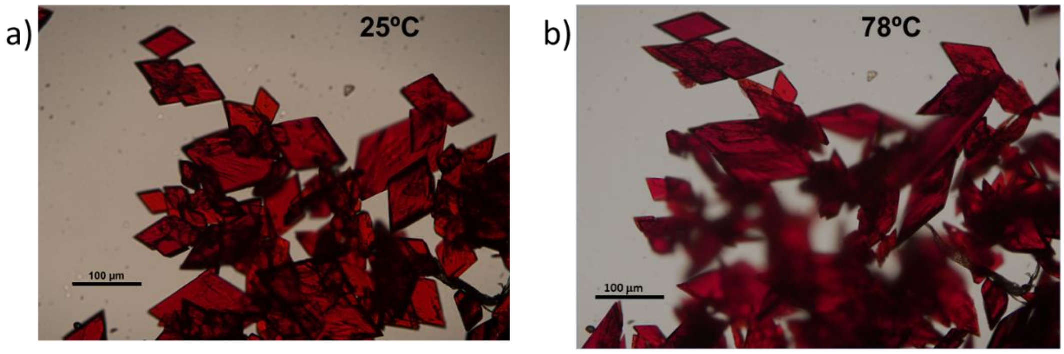

Figure S21. Thermal shape changes of bulk β phase (a) into δ phase (b).

11. Examples of previously reported thermosalient materials

Table S1. Representative reversible thermosalient organic and metaloorganic materials.

Crystal Name	Axial Thermal Expansion/shrink	Temperature of Axial Thermal Expansion	Material type	References
Ag3[Co(CN)6]	~6%	257 K	Metal complex	6
(S,S)-octa-3,5-diyn-2,7-diol 2,7-Dimethyl-octa-3,5-diyne-2,7-diol	~4% (shrink)	225 K	Organic	7
meso-tetra(4-pyridyl)porphine and CdI2	~3% (shrink)	100 K	MOF	8
Pentacene and rubrene	~1% and ~2.3% (shrink)	414 K and 100 K	Organic	9
[Ni(II)(ethylenediamine)3](oxalate anion) complex	~5% (shrink)	250K	Metal complex	10
α-(phenylazophenyl)palladium hexafluoroacetylacetonate	~4.6%	358 K	Metal complex	10
Cobalt(II) complex with a n-butyl group in its ligand, [Co(NO3)2(L)]	6–7%	253 K	Metal complex	11
[(H4BPTC)(azpy)2]n	-3.5%	273 K	Organic	12
[Co(II)(en)3](ox) (en = ethylenediamine) (crystal shrinks upon cooling)	-4.5% (shrink)	273 K	Metal complex	13
N-[[4-pdimethylaminophenylazo]benzoyl]-1phenylethylamine [trans-(S)-1]	4.5% (along width)	433 K	Organic	14
TIPS-pentacene	10 %	345 K	Organic	15

2,7-di([1,1′-biphenyl]-4-yl)-fluorenone molecules (4-DBpFO)	~10%	451 K	Organic	16
Organic–inorganic hybrid Cu(II) complex, bis(imidazolium) tetrachlorocuprate, (Himd)2[CuCl4]	~10%	393 K	Metal complex With organic salt	17
Naphthalenediimide (NDI) systems (crystal shrinks upon cooling)	-10 % (shrink)	318 K	Organic	18
Guanidinium nitrate	51.8%	310 K	Organic Salt	19
4,7-Bis[5-(4-nonylphenyl)-2-thienyl]-5,6-dimethoxy-2,1,3-benzothiadiazole (PT-BTD)	30%	351 K	Organic	In this Work

12. Computational details and DFT calculations

12.1. Computational details

All the calculations were carried out at the framework of the Density Functional Theory (DFT) level using the hybrid, generalized gradient approximation (GGA) functional B3LYP[20, 21], together with the 6-31G** basis set[22, 23]as implemented in the GAUSSIAN16 program[24]. In order to explore the impact of long-range corrections on the optical properties of these systems, the ωB97X-D functional was also used.[25]

Firstly, a preliminary study of the dihedral potential energy hypersurface between the BTD unit and the adjacent thiophene rings (see Figure 5b in the manuscript) as well as between the thiophene unit and the adjacent phenyl ring was carried out to better understand the conformation of this system (see Figure S15). The relaxed potential energy hypersurface was computed by constraining the angle of the selected dihedral bonds (by step of 10°) and allowing all other degrees of freedom to relax to their potential energy minima. The resulting potential energy curve was then referenced to the minimal energy structure.

Then, the molecular geometry was fully optimized where all geometrical parameters were allowed to vary independently. Harmonic frequencies calculations were computed, and no imaginary frequencies were observed, which ensures the finding of the global minimum energy. In order to reduce the computational cost, the alkyl chains were replaced by methyl groups.

Vertical electronic excitation energies were calculated (at the ωB97X-D/6-31G** level) by using the time-dependent DFT (TD-DFT)[26, 27] approach on the resulted molecular geometries. Absorption spectra were simulated through convolution of the vertical transition energies and oscillator strengths with Gaussian functions (half width at half-maximum of 0.3 eV).

The simulated Raman spectrum was calculated at the B3LYP/6-31G** level, for a previously optimized molecule at the same level of theory. In this case, hexyl chains were considered in order to capture the role of long alkyl chains in Raman interpretation. Raman frequencies were scaled down by a factor of 0.975, as recommended by Scott and Random[28]. The theoretical spectra were obtained by convolution the scaled frequencies and the Raman activities with Gaussian functions (5 cm^{-1} widths at the half-height).

Molecular orbitals distribution and vibrational eigenvectors were plotted using the Chemcraft 1.8 molecular modelling software.[29]

12.2. DFT calculations

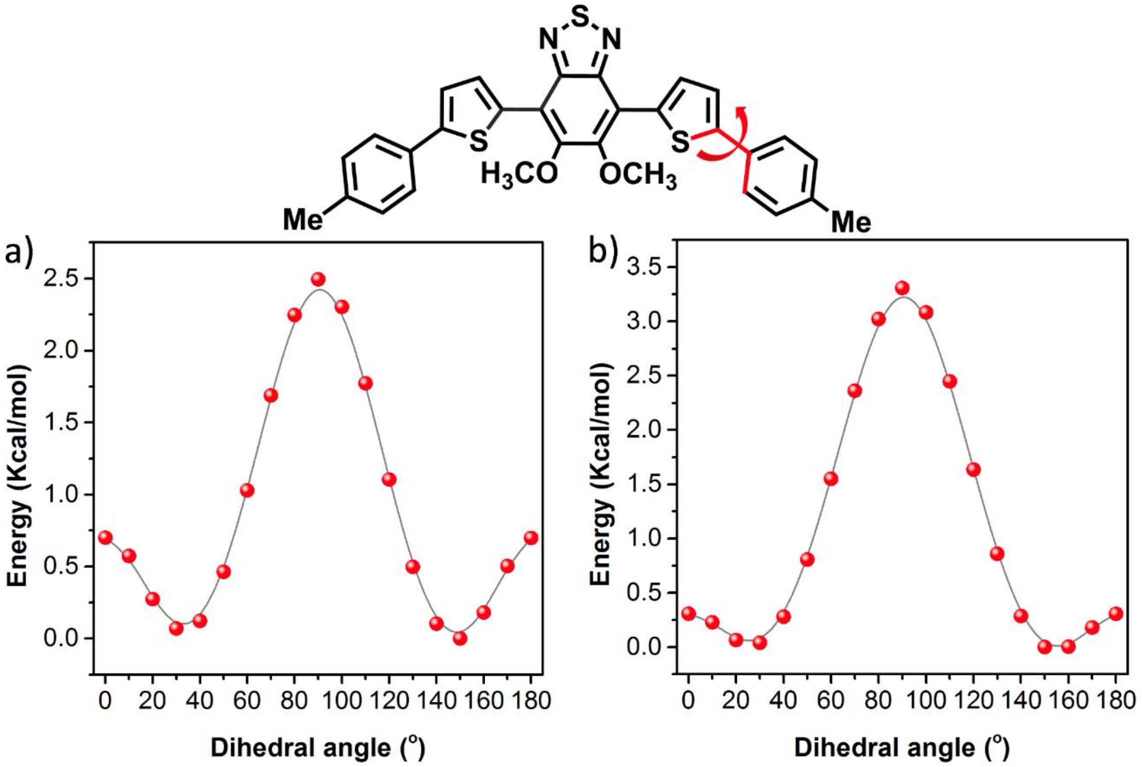

Figure S22. Dihedral potential energy surface between the thiophene unit and the adjacent phenyl ring calculated for an isolated molecule at the ωB97X-D/6-31G** (left) and B3LYP/6-31G** (right) level of theory.

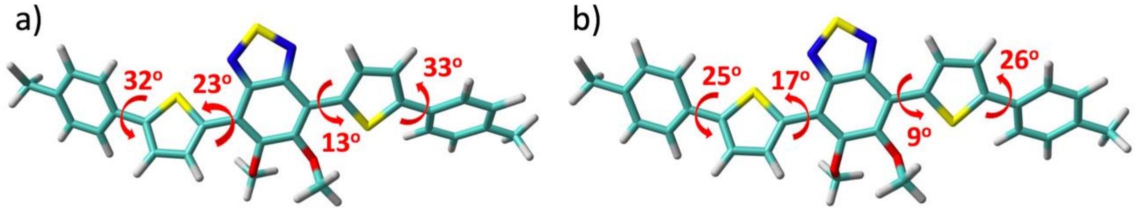

Figure S23. DFT-optimized molecular geometry for an isolated **PT-BTD** molecule at the ωB97X-D/6-31G** (a) and B3LYP/6-31G** (b) level of theory, respectively.

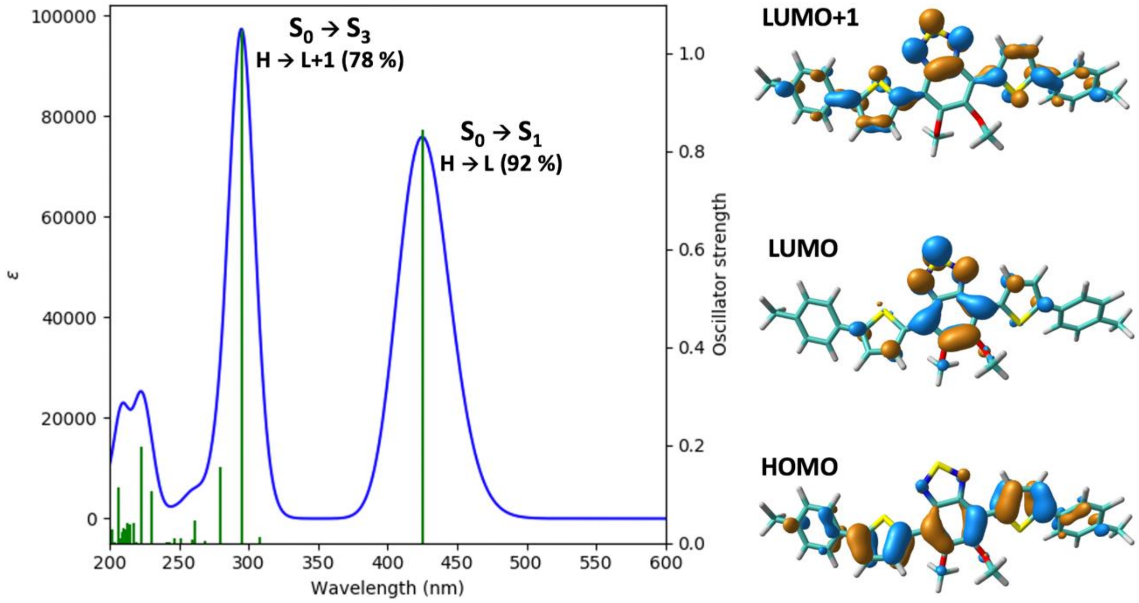

Figure S24. DFT-calculated absorption spectra at the ωB97X-D/6-31G**level for **PT-BTD** (left) and topologies of the frontier molecular orbitals involved in the two main electronic transitions calculated at the same level of theory (right).

13. Experimental and theoretical Raman spectra

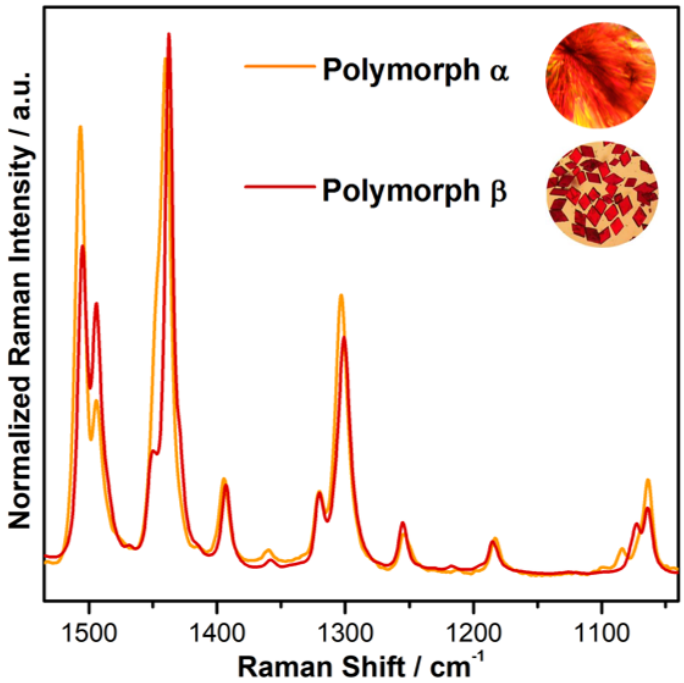

Figure S25. Comparison between Raman spectra of polymorph α (orange line) and polymorph β (red line) at 785 nm excitation.

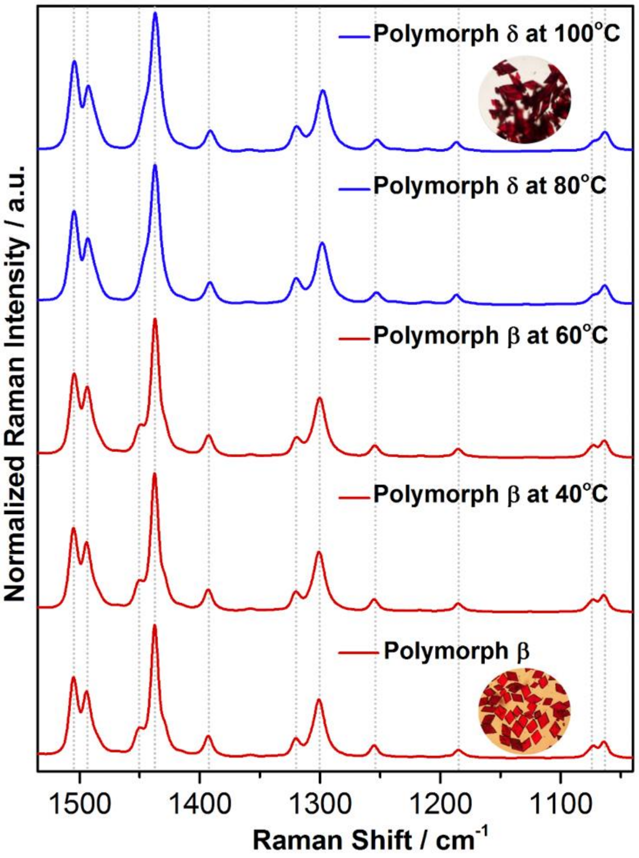

Figure S26. Thermal transformation of polymorph β (red lines) to polymorph δ (blue lines) followed by Raman spectroscopy at 785 nm excitation.

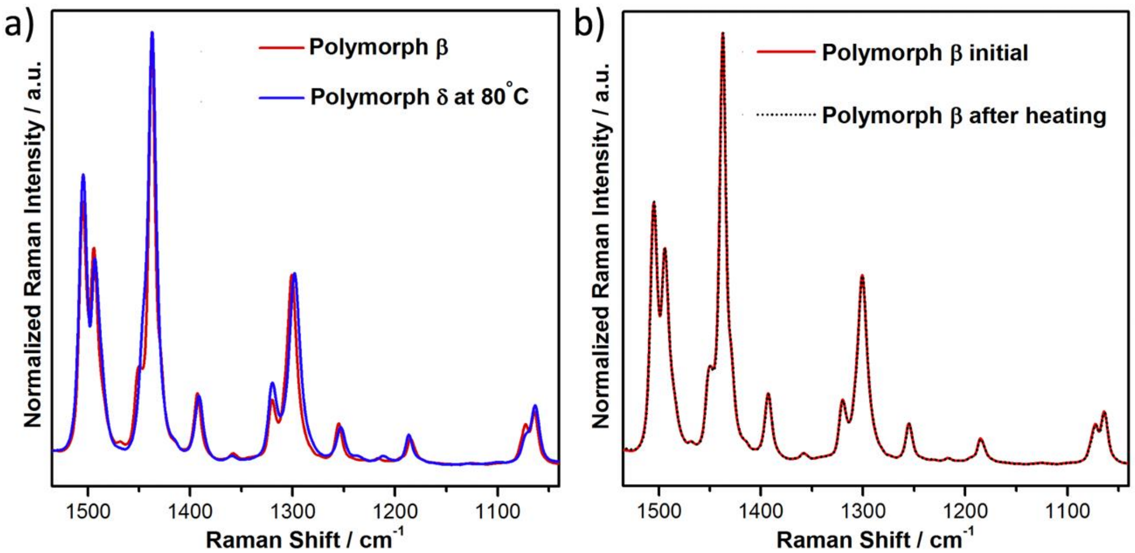

Figure S27. a) Comparison between Raman spectra of polymorph β (red line) and polymorph δ (blue line), as well as b) between Raman spectra of polymorph β (red line) and that measured after heating this phase up to 100°C (leading to polymorph δ) and then cooling to RT. Raman spectra have been collected by using a laser excitation line of 785 nm.

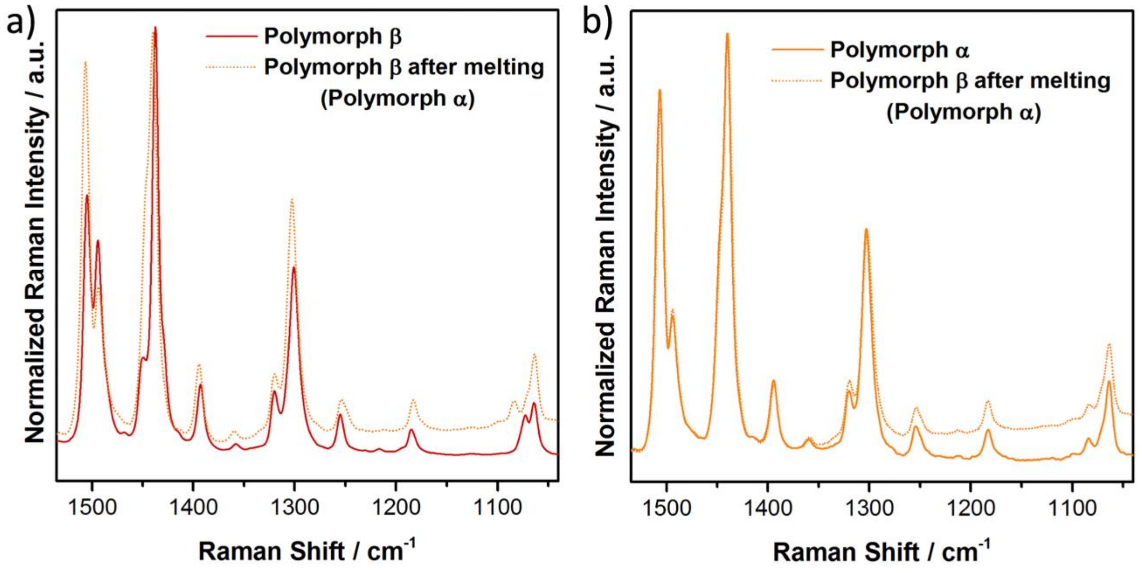

Figure S28. a) Comparison between Raman spectra of polymorph β before (red line) and after melting and then cooling to RT (dash orange line). b) Comparison between Raman spectra of isolated polymorph α and that generated after melting polymorph β and then cooling to RT. Raman spectra have been collected by using a laser excitation line of 785 nm.

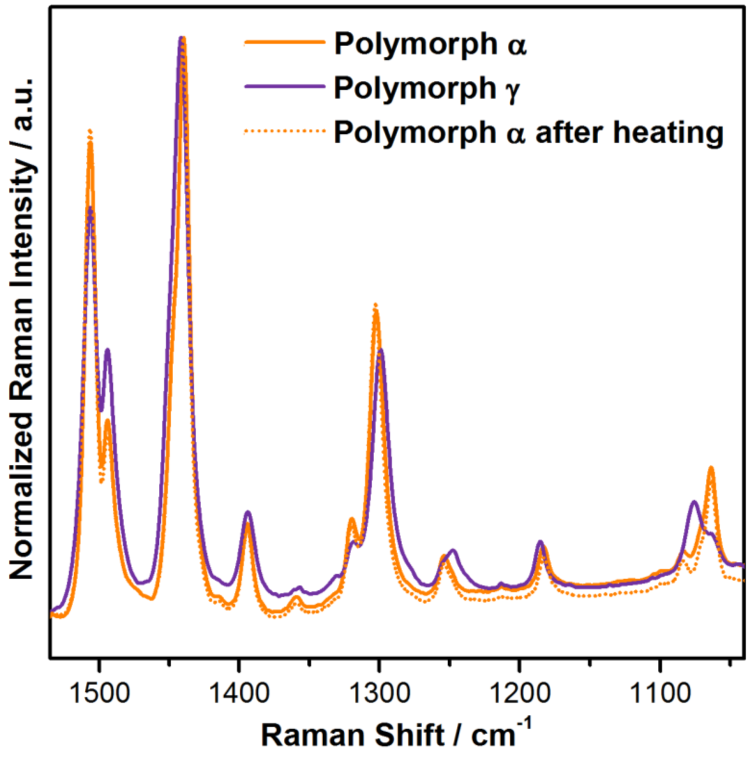

Figure S29. Comparison between Raman spectra of polymorph α (solid orange line) and that measured after heating this phase up to 105ºC (leading to polymorph γ). The Raman spectrum of polymorph α after cooling to RT (dotted orange line) is also shown. Raman spectra have been collected by using a laser excitation line of 785 nm.

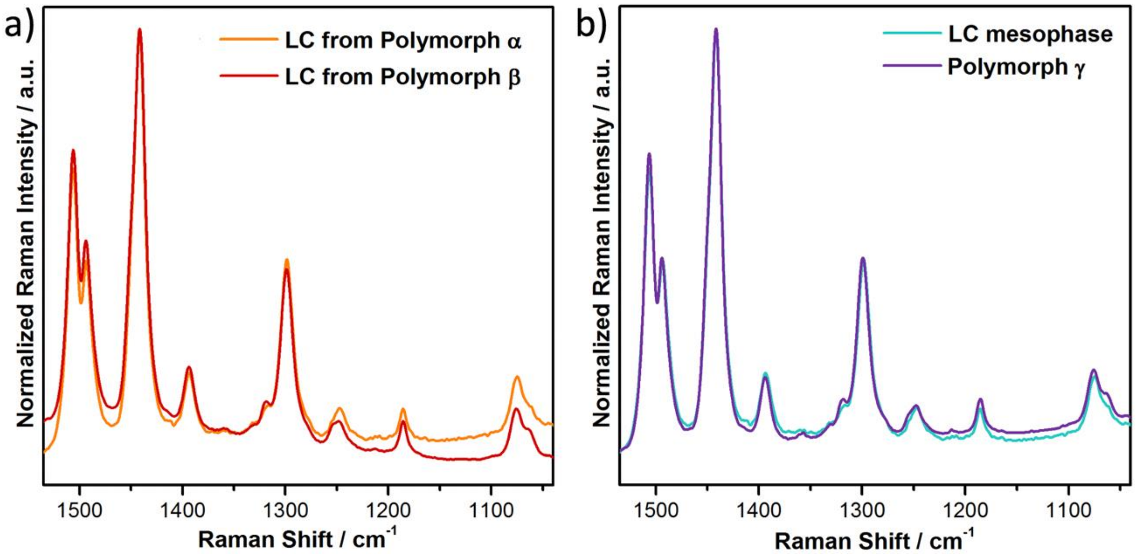

Figure S30. a) Comparison between Raman spectra of **LC** mesophase obtained from polymorph **α** (orange line) and polymorph **β** (red line). b) Comparison between the Raman spectra of **LC** mesophase (cyan line) and polymorph **γ** (purple line). Raman spectra have been collected by using a laser excitation line of 785 nm.

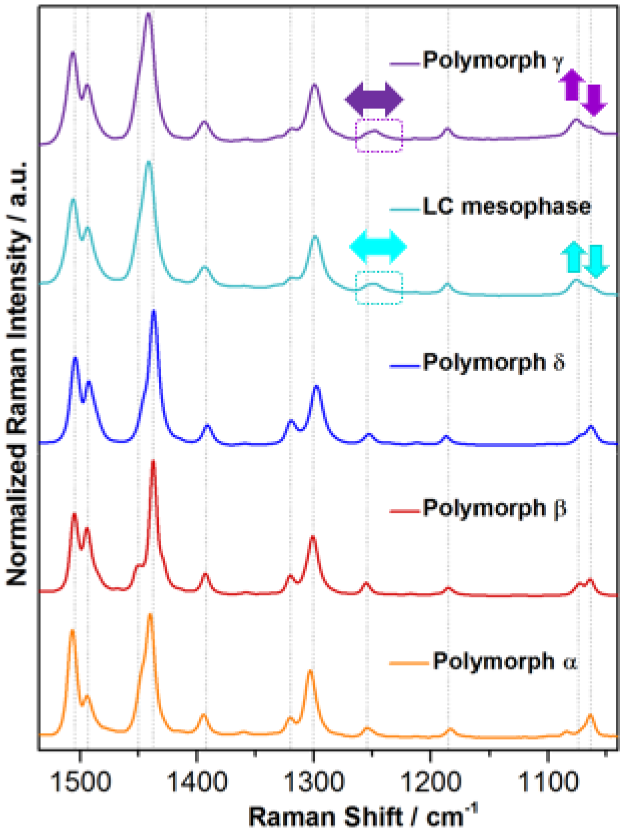

Figure S31. Comparison between the Raman spectra of all the studied phases collected at 785 nm excitation.

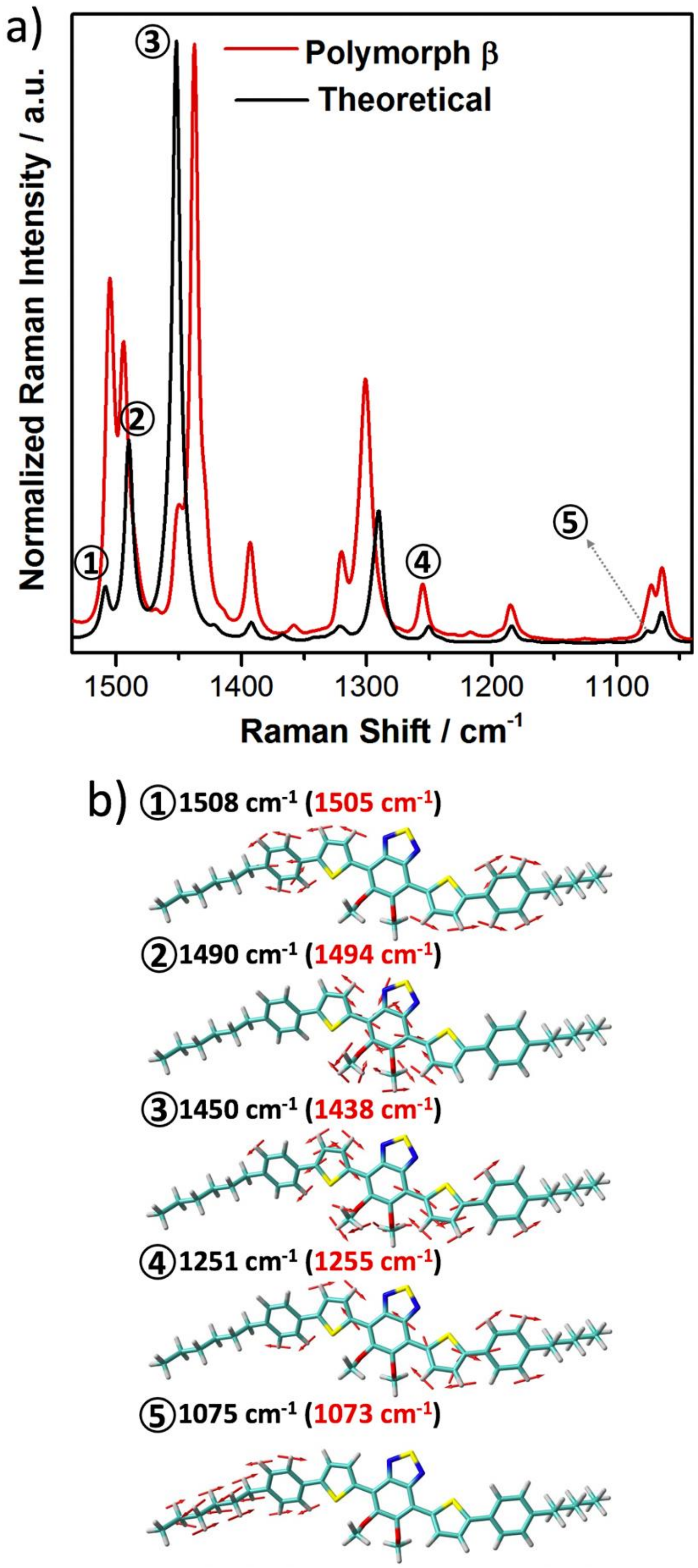

Figure S32. a) DFT-calculated Raman spectrum of **PT-BTD** at the B3LYP/6-31G** level, and b) vibrational eigenvectors associated with the most outstanding C=C/C-C Raman features. The theoretical and experimental (in parentheses) wavenumbers for polymorph β are also shown.

In an attempt to deeper insight into the molecular arrangement of these complex polymorphic transformations and considering the great utility of Raman spectroscopy to provide precise structural information in terms of π-electrons delocalization degree,[30-33] supramolecular arrangement [34-39] and for tracing polymorphic transitions [40-42] of π-conjugated systems, we have collected the Raman spectra of polymorphs α and β using a laser excitation line of 785 nm (see Figure S25). The main spectral changes can be described as following: (i) The Raman band localized at 1450 cm^{-1}, which is assigned to a collective C−C/C=C stretching vibration mainly involving the thiophene rings and the BTD unit, downshifts 5 cm^{-1} going from polymorph α to β as a consequence of a less twisted dihedral angles between the two thiophene rings with respect to the BTD unit in the latter (17° and 19° for polymorph α *vs* 14° and 8° and 15 ° and 6° for each of the two independent units in polymorph β, as shown in Figure 5a of the manuscript). (ii) A reduction in the intensity of C-C and C-N stretching vibrations localized in BTD unit (1494 cm^{-1}) is found in polymorph α; this can be ascribed to the strong proximity of the BTD groups in their supramolecular arrangement which give rise to tight S-N interactions.

Temperature dependent Raman analysis allowed us to follow the thermal expansion/compression of the crystals during the conversion from polymorph β to polymorph δ (Figure S26). This dynamic interconversion is achieved when increasing the temperature over 80 °C, and very similar spectral profiles are found in agreement with the similar molecular backbone conformation and intermolecular interactions adopted by the molecules in the two polymorphs β and δ. In addition, Raman spectroscopy confirms the complete structural reversibility of the thermal expansion/compression of the crystals, where polymorph β is regained upon cooling polymorph δ (Figure S27b). Interestingly, Raman analysis also confirms that if polymorph β is melted and then cooled to RT, it gives back polymorph α (see Figure S28).

We now turn our attention to characterize polymorph γ and **LC** mesophase. Figure S29 compares the Raman spectrum of polymorph α and that measured after heating this phase up to 105°C, which leads to polymorph γ. Interestingly, when polymorph γ is cooled back to room temperature, polymorph α is recovered. On the other hand, Figure S30a shows the Raman spectra of the LC mesophase obtained by heating polymorph α and polymorph β up to 130°C, respectively. The practically superimposable spectral profile of polymorph γ and that of the **LC** mesophase suggests similar molecular backbone conformations and alkyl chain arrangements in both cases (see Figure S30b).

Figure S31 collects the Raman spectra for all the characterized phases. A similar displacement of the CC stretching band observed at 1450 cm^{-1} is found for **LC** mesophase and polymorph γ with respect that of polymorph α; this suggests that both phases have similar intramolecular distortions. However, in comparison with the other polymorphs, the following spectral changes are observed in **LC** mesophase and polymorph γ: (i) a widening of the band around 1250 cm^{-1}, associated to inter-ring C-C stretching bonds, is found in polymorph γ and **LC** phases pointing out to an increased conformational

disorder. (ii) A change in the intensity ratio between the peaks located at 1064 and 1073 cm^{-1}, which are assigned to the trans ($\nu(C\text{–}C)_T$) and gauche ($\nu(C\text{–}C)_G$) isomers in the alkyl chains, respectively. These modes have been extensively investigated in alkane systems [43-45], and it has been found that an increase in the intensity ratio between the two peaks illustrates an increased dynamic disorder. Based on these observations, we hypothesize that the transformation taking place in **LC** mesophase and polymorph **γ** implies a decreased order of the alkyl side chains.

Finally, it is important to note that the validity of our discussion is supported by the vibrational eigenvectors and the theoretical Raman spectrum (Figure S32), which is in good agreement with the experimental data.

14. References

1. Cevher, S. C.; Hizalan, G.; Alemdar Yilmaz, E.; Cevher, D.; Udum Arslan, Y.; Toppare, L.; Yıldırım, E.; Cirpan, A., A comprehensive study: Theoretical and experimental investigation of heteroatom and substituent effects on frontier orbitals and polymer solar cell performances. *Journal of Polymer Science* **2020,** *58* (19), 2792-2806.
2. Boivin, L.-P.; Dupont, W.; Leclerc, M.; Gendron, D., Biosourced Vanillin-Based Building Blocks for Organic Electronic Materials. *The Journal of Organic Chemistry* **2021,** *86* (23), 16548-16557.
3. Apex 3; v 2019.1-0; Bruker AXS Inc.: Madison, W., USA, 2016.
4. Sheldrick, G. M., Crystal structure refinement with SHELXL. *Acta Crystallographica Section C: Structural Chemistry* **2015,** *71* (1), 3-8.
5. Dolomanov, O. V.; Bourhis, L. J.; Gildea, R. J.; Howard, J. A.; Puschmann, H., OLEX2: a complete structure solution, refinement and analysis program. *Journal of applied crystallography* **2009,** *42* (2), 339-341.
6. Goodwin, A. L.; Calleja, M.; Conterio, M. J.; Dove, M. T.; Evans, J. S.; Keen, D. A.; Peters, L.; Tucker, M. G., Colossal positive and negative thermal expansion in the framework material Ag3 [Co (CN) 6]. *Science* **2008,** *319* (5864), 794-797.
7. Das, D.; Jacobs, T.; Barbour, L. J., Exceptionally large positive and negative anisotropic thermal expansion of an organic crystalline material. *Nature Materials* **2010,** *9* (1), 36-39.
8. DeVries, L. D.; Barron, P. M.; Hurley, E. P.; Hu, C.; Choe, W., "Nanoscale lattice fence" in a metal–organic framework: interplay between hinged topology and highly anisotropic thermal response. *Journal of the American Chemical Society* **2011,** *133* (38), 14848-14851.
9. Yao, Z.-S.; Mito, M.; Kamachi, T.; Shiota, Y.; Yoshizawa, K.; Azuma, N.; Miyazaki, Y.; Takahashi, K.; Zhang, K.; Nakanishi, T., Molecular motor-driven abrupt anisotropic shape change in a single crystal of a Ni complex. *Nature Chemistry* **2014,** *6* (12), 1079-1083.
10. Panda, M. K.; Runčevski, T.; Chandra Sahoo, S.; Belik, A. A.; Nath, N. K.; Dinnebier, R. E.; Naumov, P., Colossal positive and negative thermal expansion and thermosalient effect in a pentamorphic organometallic martensite. *Nature communications* **2014,** *5* (1), 4811.
11. Su, S.-Q.; Kamachi, T.; Yao, Z.-S.; Huang, Y.-G.; Shiota, Y.; Yoshizawa, K.; Azuma, N.; Miyazaki, Y.; Nakano, M.; Maruta, G., Assembling an alkyl rotor to access

abrupt and reversible crystalline deformation of a cobalt (II) complex. *Nature communications* **2015,** *6* (1), 8810.
12. Huang, Y. G.; Shiota, Y.; Su, S. Q.; Wu, S. Q.; Yao, Z. S.; Li, G. L.; Kanegawa, S.; Kang, S.; Kamachi, T.; Yoshizawa, K., Thermally Induced Intra-Carboxyl Proton Shuttle in a Molecular Rack-and-Pinion Cascade Achieving Macroscopic Crystal Deformation. *Angewandte Chemie* **2016,** *128* (47), 14848-14852.
13. Yao, Z. S.; Wu, S. Q.; Kitagawa, Y.; Su, S. Q.; Huang, Y. G.; Li, G. L.; Ni, Z. H.; Nojiri, H.; Shiota, Y.; Yoshizawa, K., Anisotropic change in the magnetic susceptibility of a dynamic single crystal of a cobalt (II) complex. *Angewandte Chemie International Edition* **2017,** *56* (3), 717-721.
14. Taniguchi, T.; Sugiyama, H.; Uekusa, H.; Shiro, M.; Asahi, T.; Koshima, H., Walking and rolling of crystals induced thermally by phase transition. *Nature communications* **2018,** *9* (1), 538.
15. Chung, H.; Dudenko, D.; Zhang, F.; D'avino, G.; Ruzié, C.; Richard, A.; Schweicher, G.; Cornil, J.; Beljonne, D.; Geerts, Y., Rotator side chains trigger cooperative transition for shape and function memory effect in organic semiconductors. *Nature communications* **2018,** *9* (1), 278.
16. Duan, Y.; Semin, S.; Tinnemans, P.; Cuppen, H.; Xu, J.; Rasing, T., Robust thermoelastic microactuator based on an organic molecular crystal. *Nature Communications* **2019,** *10* (1), 4573.
17. Yao, Z.-S.; Guan, H.; Shiota, Y.; He, C.-T.; Wang, X.-L.; Wu, S.-Q.; Zheng, X.; Su, S.-Q.; Yoshizawa, K.; Kong, X., Giant anisotropic thermal expansion actuated by thermodynamically assisted reorientation of imidazoliums in a single crystal. *Nature communications* **2019,** *10* (1), 4805.
18. Dharmarwardana, M.; Pakhira, S.; Welch, R. P.; Caicedo-Narvaez, C.; Luzuriaga, M. A.; Arimilli, B. S.; McCandless, G. T.; Fahimi, B.; Mendoza-Cortes, J. L.; Gassensmith, J. J., Rapidly reversible organic crystalline switch for conversion of heat into mechanical energy. *Journal of the American Chemical Society* **2021,** *143* (15), 5951-5957.
19. Karothu, D. P.; Ferreira, R.; Dushaq, G.; Ahmed, E.; Catalano, L.; Halabi, J. M.; Alhaddad, Z.; Tahir, I.; Li, L.; Mohamed, S., Exceptionally high work density of a ferroelectric dynamic organic crystal around room temperature. *Nature communications* **2022,** *13* (1), 2823.
20. Lee, C.; Yang, W.; Parr, R. G., Development of the Colle-Salvetti correlation-energy formula into a functional of the electron density. *Phys. Rev. B* **1988,** *37* (2), 785-789.
21. Becke, A. D., Density-functional thermochemistry. III. The role of exact exchange. *J. Chem. Phys.* **1993,** *98* (7), 5648-5652.
22. Hehre, W. J.; Ditchfield, R.; Pople, J. A., Self—Consistent Molecular Orbital Methods. XII. Further Extensions of Gaussian—Type Basis Sets for Use in Molecular Orbital Studies of Organic Molecules. *J. Chem. Phys.* **1972,** *56* (5), 2257-2261.
23. Francl, M. M.; Pietro, W. J.; Hehre, W. J.; Binkley, J. S.; Gordon, M. S.; DeFrees, D. J.; Pople, J. A., Self-consistent molecular orbital methods. XXIII. A polarization-type basis set for second-row elements. *The Journal of Chemical Physics* **1982,** *77* (7), 3654-3665.
24. M. J. Frisch, G. W. T., H. B. Schlegel, G. E. Scuseria, M. A. Robb, J. R. Cheeseman, G. Scalmani, V. Barone, G. A. Petersson, H. Nakatsuji, X. Li, M. Caricato, A. V. Marenich, J. Bloino, B. G. Janesko, R. Gomperts, B. Mennucci, H. P. Hratchian, J. V. Ortiz, A. F. Izmaylov, J. L. Sonnenberg, D. Williams-Young, F. Ding, F. Lipparini, F. Egidi, J. Goings, B. Peng, A. Petrone, T. Henderson, D. Ranasinghe, V. G. Zakrzewski,

J. Gao, N. Rega, G. Zheng, W. Liang, M. Hada, M. Ehara, K. Toyota, R. Fukuda, J. Hasegawa, M. Ishida, T. Nakajima, Y. Honda, O. Kitao, H. Nakai, T. Vreven, K. Throssell, J. A. Montgomery, Jr., J. E. Peralta, F. Ogliaro, M. J. Bearpark, J. J. Heyd, E. N. Brothers, K. N. Kudin, V. N. Staroverov, T. A. Keith, R. Kobayashi, J. Normand, K. Raghavachari, A. P. Rendell, J. C. Burant, S. S. Iyengar, J. Tomasi, M. Cossi, J. M. Millam, M. Klene, C. Adamo, R. Cammi, J. W. Ochterski, R. L. Martin, K. Morokuma, O. Farkas, J. B. Foresman, and D. J. Fox. , Gaussian 16, Revision B.01. *Gaussian, Inc., Wallingford CT,* **2016**.
25. Chai, J.-D.; Head-Gordon, M., Long-range corrected hybrid density functionals with damped atom–atom dispersion corrections. *Phys. Chem. Chem. Phys.* **2008,** *10* (44), 6615-6620.
26. Runge, E.; Gross, E. K. U., Density-Functional Theory for Time-Dependent Systems. *Phys. Rev. Lett.* **1984,** *52* (12), 997-1000.
27. Heinze, H. H.; Görling, A.; Rösch, N., An efficient method for calculating molecular excitation energies by time-dependent density-functional theory. *J. Chem. Phys.* **2000,** *113* (6), 2088-2099.
28. Scott, A. P.; Radom, L., Harmonic Vibrational Frequencies: An Evaluation of Hartree−Fock, Møller−Plesset, Quadratic Configuration Interaction, Density Functional Theory, and Semiempirical Scale Factors. *J. Phys. Chem.* **1996,** *100* (41), 16502-16513.
29. https://www.chemcraftprog.com, Chemcraft - graphical software for visualization of quantum chemistry computations. .

.
30. Gámez-Valenzuela, S.; Benito-Hernández, A.; Echeverri, M.; Gutierrez-Puebla, E.; Ponce Ortiz, R.; Ruiz Delgado, M. C.; Gómez-Lor, B., Functionalized Crystalline N-Trimethyltriindoles: Counterintuitive Influence of Peripheral Substituents on Their Semiconducting Properties. *Molecules* **2022,** *27* (3), 1121.
31. Ruiz, C.; López Navarrete, J. T.; Ruiz Delgado, M. C.; Gómez-Lor, B., Triindole-Bridge-Triindole Dimers as Models for Two Dimensional Microporous Polymers. *Org. Lett.* **2015,** *17* (9), 2258-2261.
32. Ruiz, C.; Arrechea-Marcos, I.; Benito-Hernández, A.; Gutierrez-Puebla, E.; Monge, M. A.; López Navarrete, J. T.; Ruiz Delgado, M. C.; Ortiz, R. P.; Gómez-Lor, B., Solution-processed N-trialkylated triindoles for organic field effect transistors. *J. Mater. Chem. C* **2018,** *6* (1), 50-56.
33. Wang, Y.; Guo, H.; Ling, S.; Arrechea-Marcos, I.; Wang, Y.; López Navarrete, J. T.; Ortiz, R. P.; Guo, X., Ladder-type Heteroarenes: Up to 15 Rings with Five Imide Groups. *Angew. Chem. Int. Ed.* **2017,** *56* (33), 9924-9929.
34. Echeverri, M.; Ruiz, C.; Gámez-Valenzuela, S.; Martín, I.; Ruiz Delgado, M. C.; Gutiérrez-Puebla, E.; Monge, M. Á.; Aguirre-Díaz, L. M.; Gómez-Lor, B., Untangling the Mechanochromic Properties of Benzothiadiazole-Based Luminescent Polymorphs through Supramolecular Organic Framework Topology. *J. Am. Chem. Soc.* **2020,** *142* (40), 17147-17155.
35. Echeverri, M.; Ruiz, C.; Gámez-Valenzuela, S.; Alonso-Navarro, M.; Gutierrez-Puebla, E.; Serrano, J. L.; Ruiz Delgado, M. C.; Gómez-Lor, B., Stimuli-Responsive Benzothiadiazole Derivative as a Dopant for Rewritable Polymer Blends. *ACS Appl. Mater. Interfaces* **2020,** *12* (9), 10929-10937.
36. Milani, A.; Brambilla, L.; Del Zoppo, M.; Zerbi, G., Raman Dispersion and Intermolecular Interactions in Unsubstituted Thiophene Oligomers. *J. Phys. Chem. B* **2007,** *111* (6), 1271-1276.

37. Sainbileg, B.; Lan, Y.-B.; Wang, J.-K.; Hayashi, M., Deciphering Anomalous Raman Features of Regioregular Poly(3-hexylthiophene) in Ordered Aggregation Form. *J. Phys. Chem. C* **2018,** *122* (8), 4224-4231.
38. Mosca, S.; Milani, A.; Peña-Álvarez, M.; Yamaguchi, S.; Hernández, V.; Ruiz Delgado, M. C.; Castiglioni, C., Mechanochromic Luminescent Tetrathiazolylthiophenes: Evaluating the Role of Intermolecular Interactions through Pressure and Temperature-Dependent Raman Spectroscopy. *J. Phys. Chem. C* **2018,** *122* (30), 17537-17543.
39. Milani, A.; Del Zoppo, M.; Tommasini, M.; Zerbi, G., The Effect of Intermolecular Dipole−Dipole Interaction on Raman Spectra of Polyconjugated Molecules: Density Functional Theory Simulations and Mathematical Models. *J. Phys. Chem. B* **2008,** *112* (6), 1619-1625.
40. Bi, D.; Henkes, S.; Daniels, K. E.; Chakraborty, B., The statistical physics of athermal materials. *Annu. Rev. Condens. Matter Phys.* **2015,** *6* (1), 63-83.
41. Tandon, P.; Förster, G.; Neubert, R.; Wartewig, S., Phase transitions in oleic acid as studied by X-ray diffraction and FT-Raman spectroscopy. *Journal of Molecular Structure* **2000,** *524* (1-3), 201-215.
42. Wood, S.; Rigas, G.-P.; Zoladek-Lemanczyk, A.; Blakesley, J. C.; Georgakopoulos, S.; Mas-Torrent, M.; Shkunov, M.; Castro, F. A., Precise characterisation of molecular orientation in a single crystal field-effect transistor using polarised Raman spectroscopy. *Scientific reports* **2016,** *6* (1), 33057.
43. Orendorff, C. J.; Ducey Jr, M. W.; Pemberton, J. E., Quantitative correlation of Raman spectral indicators in determining conformational order in alkyl chains. *The Journal of Physical Chemistry A* **2002,** *106* (30), 6991-6998.
44. Scherrer, D.; Vogel, D.; Drechsler, U.; Olziersky, A.; Sparr, C.; Mayor, M.; Lörtscher, E., Monitoring Solid-Phase Reactions in Self-Assembled Monolayers by Surface-Enhanced Raman Spectroscopy. *Angewandte Chemie International Edition* **2021,** *60* (33), 17981-17988.
45. Ando, M.; Yoneya, M.; Kehoe, T. B.; Ishii, H.; Minakata, T.; Kawasaki, M.; Duffy, C. M.; Phillips, R.; Sirringhaus, H., Disorder and localization dynamics in polymorphs of the molecular semiconductor pentacene probed by in situ micro-Raman spectroscopy and molecular dynamics simulations. *Physical Review Materials* **2019,** *3* (2), 025601.
